# A phase IA dose-escalation study of PHI-101, a new checkpoint kinase 2 inhibitor, for platinum-resistant recurrent ovarian cancer

**DOI:** 10.1186/s12885-021-09138-z

**Published:** 2022-01-03

**Authors:** Soo Jin Park, Suk-Joon Chang, Dong Hoon Suh, Tae Wook Kong, Heekyoung Song, Tae Hun Kim, Jae-Weon Kim, Hee Seung Kim, Sung-Jong Lee

**Affiliations:** 1 Department of Obstetrics and Gynecology, Seoul National University College of Medicine, 101 Daehak-Ro, Jongno-Gu, Seoul, 03080 Republic of Korea; 2 Gynecologic Cancer Center, Department of Obstetrics and Gynecology, Ajou University School of Medicine, Suwon, Republic of Korea; 3 Department of Obstetrics and Gynecology, Seoul National University Bundang Hospital, Seoul National University College of Medicine, Seongnam, Republic of Korea; 4 Department of Obstetrics and Gynecology, Seoul St. Mary’s Hospital, College of Medicine, The Catholic University of Korea, 222 Banpo-daero Seocho-gu, Seoul, 06591 Republic of Korea; 5 Department of Obstetrics and Gynecology, Seoul Metropolitan Government Seoul National University Boramae Medical Center, Seoul, Korea

**Keywords:** Platinum-resistance, ovarian cancer, Chk2 inhibitor, PARP inhibitor, Phase IA

## Abstract

**Background:**

PHI-101 is an orally available, selective checkpoint kinase 2 (Chk2) inhibitor. PHI-101 has shown anti-tumour activity in ovarian cancer cell lines and impaired DNA repair pathways in preclinical experiments. Furthermore, the in vivo study suggests the synergistic effect of PHI-101 through combination with PARP inhibitors for ovarian cancer treatment. The primary objective of this study is to evaluate the safety and tolerability of PHI-101 in platinum-resistant recurrent ovarian cancer.

**Methods:**

Chk2 inhibitor for Recurrent EpitheliAl periToneal, fallopIan, or oVarian cancEr (CREATIVE) trial is a prospective, multi-centre, phase IA dose-escalation study. Six cohorts of dose levels are planned, and six to 36 patients are expected to be enrolled in this trial.

Major inclusion criteria include ≥ 19 years with histologically confirmed epithelial ovarian cancer, fallopian tube carcinoma, or primary peritoneal cancer. Also, patients who showed disease progression during platinum-based chemotherapy or disease progression within 24 weeks from completion of platinum-based chemotherapy will be included, and prior chemotherapy lines of more than five will be excluded. The primary endpoint of this study is to determine the dose-limiting toxicity (DLT) and maximum tolerated dose (MTD) of PHI-101.

**Discussion:**

PHI-101 is the first orally available Chk2 inhibitor, expected to show effectiveness in treating recurrent ovarian cancer. Through this CREATIVE trial, DLT and MTD of this new targeted therapy can be confirmed to find the recommended dose for the phase II clinical trial. This study may contribute to developing a new combination regimen for the treatment of ovarian cancer.

**Trial registration:**

ClinicalTrials.gov Identifier: NCT04678102.

**Supplementary Information:**

The online version contains supplementary material available at 10.1186/s12885-021-09138-z.

## Background

DNA damage repair (DDR) system impairment has been associated with ovarian cancer carcinogenesis [1]. Approximately 50% of high-grade serous ovarian cancer is associated with homologous recombination deficiency (HRD) and other DDR systems, including base excision repair, nucleotide excision repair, mismatch repair, and non-homologous end-joining system, also attributed to ovarian cancer development [1, 2]. Especially, tumours harbouring HRD showed synthetic lethality leads to higher sensitivity in poly(ADP-ribose) polymerase (PARP) inhibitors. Recent clinical trials on advanced or recurrent ovarian cancer have shown efficacy with PARP inhibitors. Olaparib increased progression-free survival in BRCA1/2 mutated patients in primary ovarian cancer [3], and niraparib prolonged progression-free survival regardless of HRD status or BRCA mutations in primary ovarian cancer [4].

Checkpoint kinase 1 and 2 (Chk1 and 2) are activated by ataxia telangiectasia mutated kinase (ATM) and ataxia telangiectasia and Rad3-related kinase (ATR) pathways, which are mainstreams of the DDR system [5]. Chk1 and 2 are activated by DNA double-strand breakage and involved in the homologous recombination (HR) pathway. ATR phosphorylates Chk1, and the function of ATR and Chk1 is essential in cell cycle regulation and the DDR system. Several Chk1 and 2 depleting agents were developed, and prexasertib, which showed higher affinity to Chk1, was one of the promising molecules [6, 7]. A phase II study of prexasertib on BRCA mutant-type recurrent ovarian cancer yielded only an 11.1% response rate and a 29% response rate in BRCA wild-type recurrent ovarian cancer [6, 8].

On the other hand, Chk2 is a serine/threonine kinase and functions as a barrier in tumorigenesis by maintaining genomic stability, and loss of Chk2 is known to be discovered in solid tumours, including ovarian cancer [9]. PHI-101 is the first oral Chk2 selective inhibitor, identified by artificial intelligence and a big data-based in-house drug discovery platform. Anti-tumour activity of PHI-101 is shown in various ovarian cancer cell lines, including CAOV3, OVCAR3, and SC-OV3. PHI-101 induced impairment of chk2 downstream DNA repair pathway and anti-proliferative activity in ovarian cancer cell lines. Patient-derived tumour spheroid culture also showed anti-cancer activity of PHI-101 regardless of BRCA1 status [10]. Thus, PHI-101, a new Chk2 inhibitor, is expected to suggest a different treatment strategy for ovarian cancer, either alone or as a combination therapy. Therefore, we designed a phase I dose-escalation trial to evaluate the safety and tolerability of PHI-101 for platinum-resistant recurrent ovarian cancer.

## Methods/Design

### Trial design

Chk2 inhibitor for Recurrent EpitheliAl periToneal, fallopIan, or oVarian cancEr (CREATIVE) trial is a prospective, multi-centre, phase I dose-escalation study to determine the dose-limiting toxicity (DLT) and maximum tolerated dose (MTD) of PHI-101. This study was approved by each institutional review board of all participating institutions and funded by Pharos iBio Co., Ltd. (Gyeonggi-do, Republic of Korea). Four participating institutions are listed: Ajou University Hospital, Bundang Seoul National University Hospital, Seoul National University Hospital, and Seoul ST. Mary's Hospital.

**Fig. 1:**
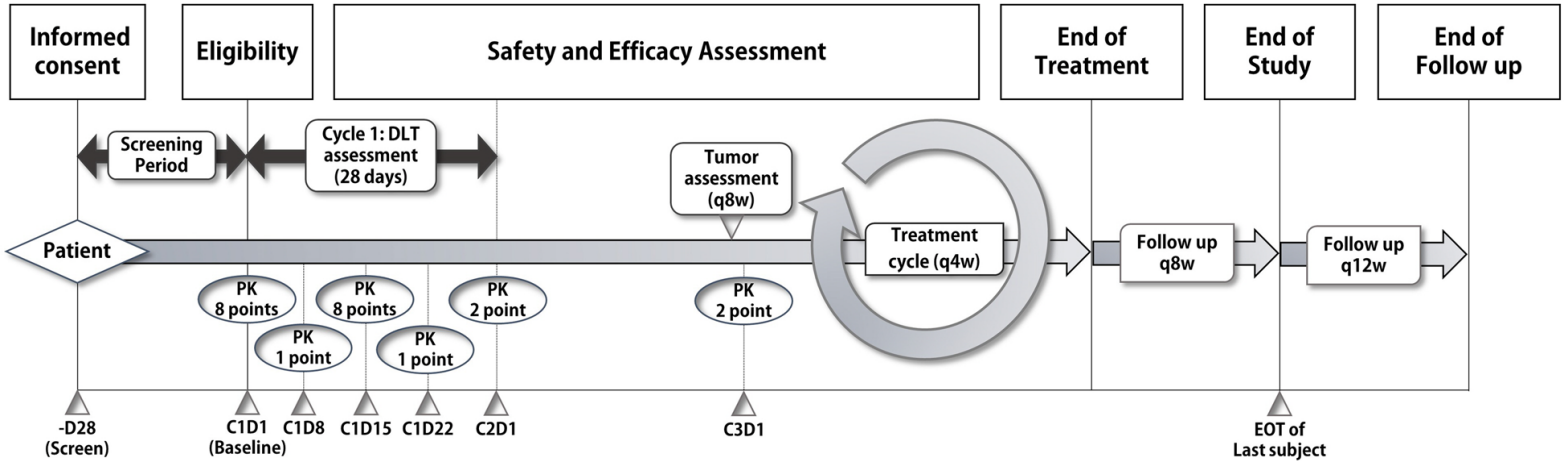
Study design schema. (Abbreviations: DLT, dose-limiting toxicity; EOT, end of treatment; PK, pharmacokinetic sampling)

The investigators will obtain the informed consent form from all participants before any screening examinations. As shown in Figure 1, study participants will be orally administered PHI-101 (2 to 12 tablets/day) at a predetermined dose cohort once daily each for one cycle (28 days), and DLT will be observed during the first cycle of each subject by the list depicted in Table 1. Participants will continue to take PHI-101 until the following termination criteria are met: radiologic progression or clinical progression; death; withdrawal of consent; unacceptable adverse event; dose interruption longer than four weeks. Dose adjustment is not allowed during the DLT observation period (cycle 1, 28 days). Although treatment-specific side effects are not known so far, we included possible side effects shown in preclinical study results depicted in Supplementary Table 2 to the DLT list. During the study period, concomitant medication to control symptoms other than tumours is permitted. However, any antineoplastic therapies other than the IP (surgery, radio(chemo)therapy, cytotoxic chemotherapy, targeted therapy, and immuno-oncologic drug) and alternative treatments (nonprescription drug, herb, or homoeopathy) will be prohibited.

**Table 1:** Definition of dose limiting toxicity (DLT)

CTCAE Term	CTCAE version 5.0
**Hematological toxicity**	
Febrile neutropenia	grade ≥ 3
Neutrophil count decreased	grade ≥ 3 for > 7 days
WBC decreased	grade ≥ 3
Platelet count decreased	grade ≥ 3
	grade < 3 requiring blood transfusion
Anemia	grade ≥ 3
**Non-hematological toxicity**	
ECG QT corrected interval prolonged	grade ≥ 3
Nausea	grade ≥ 3 for > 7 days despite the adequate and optimal therapy
Tumor pain	grade ≥ 3 for > 7 days despite the adequate and optimal therapy
Vomiting	grade 3 for > 7 days despite the adequate and optimal therapy, or grade ≥ 4
Diarrhea or associated electrolyte abnormalities	grade ≥ 3 for > 2 days despite the adequate and optimal therapy
Fatigue	grade ≥ 3 for > 7 days
Anorexia	grade ≥ 3 for > 7 days
Hypophosphatemia, hypomagnesemia, or hypocalcemia	grade ≥ 3 for > 2 days despite the adequate and optimal therapy
Asymptomatic AST, ALT, ALP, or GGT	grade ≥ 3 for > 7 days
Baseline AST or ALT ≥ 2.5 to 5 X ULN in patients with confirmed liver metastases	AST or ALT > 8 X ULN for > 7 days
Baseline ALP ≥ 2 to 5 X ULN in patients with confirmed liver metastases	ALP > 8 X ULN for > 7 days
All the other ADRs excluding above	grade ≥ 3
**Other toxicity**	
ADR with dose interruption (temporary discontinuation) of PHI-101 for > 4 weeks	

Abbreviations: ALP, alkaline phosphatase; ALT, alanine transferase; AST, aspartate transferase; CTCAE, Common Terminology Criteria for Adverse Events; ECG, electrocardiography; GGT, γ-glutamyl transferase; ULN, upper normal limit; WBC, white blood count.

**Fig. 2:**
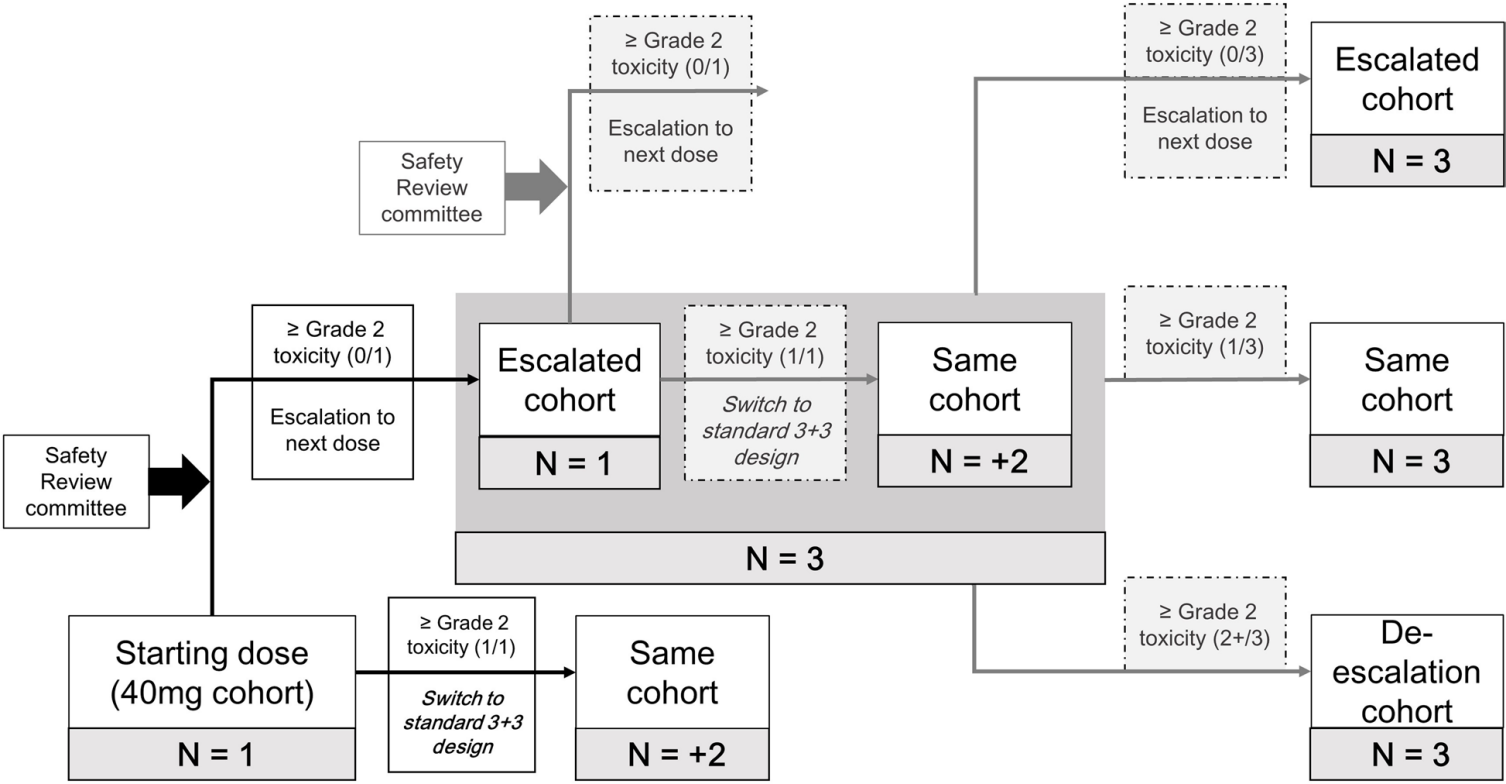
Accelerated dose escalation and switching to standard 3+3 dose escalation scheme.

The study scheme for the cohort assignment is described in Fig. 2 [11]. The accelerated dose escalation scheme, which assesses DLT in a single subject in each cohort, will be applied for this phase I study. This accelerated dose-escalation scheme will be sustained until adverse drug reaction related to investigational product (IP) same or greater than grade 2 occurs. If IP-related toxicity ≥ grade 2 does not occur, DLT can be assessed at the higher dose cohort according to the recommendation of the safety review committee (SRC). If IP-related toxicity ≥ grade 2 occurs, additional two subjects will be enrolled in the same dose cohort, and the study will be switched to the standard 3+3 scheme. If DLT is observed in > 1 out of 6 subjects in a specific cohort (χ) and DLT is observed in ≤ 1 out of 6 subjects in the cohort (χ-1) that is one level lower than the specific cohort, the one level lower cohort (χ-1) will be considered as MTD. The dose of PHI-101 will be escalated until MTD is determined, and if the MTD is not determined at the maximum planned dose, dose-escalation will be ended at that dose.

### Participants

#### Major inclusion criteria

1. Females aged ≥ 19 years
2. Histologically confirmed epithelial ovarian cancer, fallopian tube cancer, or primary peritoneal cancer regardless of initial stage at diagnosis
3. Platinum-refractory (disease progression during platinum-based chemotherapy) or platinum-resistance cancer (disease progression within 24 weeks from completion of platinum-based chemotherapy) in which patients progressed after second-line or more platinum-containing chemotherapy will be included
4. A life expectancy ≥ 12 weeks assessed by investigators comprehensively judging clinical status
5. A prior number of cytotoxic chemotherapy lines ≤ 5
6. Eastern Cooperative Oncology Group (ECOG) performance status ≤ 1

#### Major Exclusion Criteria

1. Platinum-sensitive ovarian cancer (disease progression after 24 weeks from completion of platinum-based chemotherapy)
2. Prior number of cytotoxic chemotherapy lines > 5
3. Known or suspected hypersensitivity or intolerance to the active ingredient or excipients of PHI-101
4. Patients with severe cardiovascular disease, intake or absorption disability (dysphagia, intestinal paralysis or obstruction, and history of gastrointestinal surgery significantly affect absorption), autoimmune or inflammatory disease, severe respiratory disease, active hepatitis B or C, and several infectious diseases will be excluded.
5. ECOG performance status ≥ 2

### Primary Endpoints

#### Primary objectives

The primary objective of this study is to assess DLT and MTD of PHI-101. In addition, the presented by the cohort and MTD will be determined. The definition of DLT is presented in Table 1.

#### Secondary objectives

Secondary objectives include assessing the IP tolerability by dose interruption, dose reduction, and dose termination due to adverse events. In addition, IP safety will be assessed by treatment-emergent adverse events, adverse drug reactions, serious adverse events, serious adverse drug reactions, and adverse events leading to withdrawal. Adverse events are defined as any unfavourable and unintended sign, symptom, or disease during the study period. Adverse events will be assessed according to the Common Terminology Criteria for Adverse Events (CTCAE) version 5.0 criteria. Also, physical examination, laboratory tests, vital signs, electrocardiogram will be performed for safety evaluation, and the investigators, including physicians and coordinating research nurse, will assess the adverse events for every visit.

Pharmacokinetic assessment will be done on cycle 1 day 1 (pre-dose, 0.5-, 1-, 2-, 4-, 6-, 8- and 24-hours post-dose), day 8 (pre-dose), day 15 (pre-dose, 0.5-, 1-, 2-, 4-, 6-, 8- and 24 hours post-dose), day 22 (pre-dose), cycle 2 day 1 (pre-dose and 2-4 hours post-dose), cycle 2 day 1 (pre-dose and 1-3 hours post-dose), and then every three cycles on day 1 to end of treatment (EOT). For pharmacokinetic analysis of PHI-101, 6mL of blood will be collected using a K2 EDTA tube and then centrifuged at 2,000g at 4°C for 10 minutes. The separated supernatant (plasma) will be dispensed into two tubes by 1mL or more and stored in a freezer at -70°C or lower.

Genetic variation, including HRD related genes including BRCA mutation based on tumour next-generation sequencing test results, will be collected for exploratory assessment. Additional biopsy or sequencing is not mandatory for the participants, but previously performed analysis results based on medical records will be analysed. For the efficacy assessment, a radiologic tumour response assessment will be done based on RECIST version 1.1.

The participants will be required to write a drug diary to improve adherence. Adequate and optimal supportive care will be permitted during the study, and therapies that may affect the efficacy and safety assessment of IP will be prohibited.

### Sample size

Given the characteristics of a phase I study, calculating the sample size based on a statistical hypothesis was not conducted in this study. Instead, the target number of subjects will be determined to ensure the smallest possible number of subjects participating in the study. A total of six cohorts are planned in the study, and a minimum of one to a maximum of six subjects will be enrolled in each cohort (Table 2). Therefore, approximately we expect six to 36 patients to be enrolled.

**Table 2:** Dose cohort and escalation increment

Dose level (cohort)	Daily dose of PHI-101 (once daily)		Escalation increment from the previous dose	
-1	20 mg	1 tab.	- 20 mg	- 50%
1 (starting dose)	40 mg	2 tab.	-	-
2	80 mg	4 tab.	40 mg	100%
3	120 mg	6 tab.	40 mg	50%
4	160 mg	8 tab.	40 mg	33%
5	200 mg	10 tab.	40 mg	25%
6 (maximum planned dose)	240 mg	12 tab.	40 mg	20%

### Statistical method

Safety analysis will be done on the subjects who received at least a single dose of the IP, and the number of events will be presented using the number of events by cohort. In addition, the DLT assessment will be done on the subjects who received at least a single dose of the IP and had DLT assessments during cycle 1. PK parameters including C_max_, C_max,ss_, C_min,ss_, C_av,ss_, AUC_t_, AUC_τ_, AUC_inf_, T_max_, T_max,ss_, t_1/2_, peak-trough fluctuation (PTF), accumulation ratio (AR), CL/F, CL_ss_/F, and V_z_/F will be calculated.

### Data monitoring and management

The SRC will periodically review the adverse events and risk assessment. SRC consists of the coordinating investigator, the principal investigators, the sponsor, and medical advisors. Data will be recorded in the electronic case report form (e-CRF), and only authorised personnel can access the data. The sponsor may conduct audits to ensure that the study is conducted in compliance with the International Council for Harmonisation of Technical Requirements for Pharmaceuticals for Human Use-Good Clinical Practice (ICH-GCP), Korea Good Clinical Practice (KGCP), and basic principles of the Declaration of Helsinki.

Protocol amendment is not permitted once after study initiation without the consent of the other. Once initiated, amendments can be made only in the exceptional case, and all involved parties must provide a written consent form.

## Discussion

PHI-101 is the first orally available and a selective inhibitor of Chk2. *In vivo* and *ex vivo* experimental results imply that PHI-101 may allow ovarian cancer cells to obtain synthetic lethality, especially when combined with PARP inhibitors [10]. In detail, PHI-101 and olaparib showed a synergistic effect in ovarian and breast cancer cell lines, regardless of BRCA and p53 expression status [10]. Therefore, Chk2 inhibitor is expected to be a new treatment strategy for ovarian cancer, either alone or in combination with PARP inhibitors.

Molecular characteristics such as HRD or p53 alteration of ovarian cancer tumours allow high sensitivity to cytotoxic chemotherapy, anti-angiogenetic agent, and PARP inhibitors, a part of molecular deficiencies is known to be restored in some platinum-resistant recurrent ovarian cancers. Increasing DNA repair and restoration of HR repair are known to be one of the mechanisms of platinum-resistance in recurrent ovarian cancer [12, 13]. To overcome platinum-resistance, several new combinations with targeted therapy or immune checkpoint blockade agents are investigated [14, 15]. Still, only bevacizumab has been shown to improve progression-free survival in platinum-resistant ovarian cancer [15]. As PARP inhibitors or anti-angiogenic agents are now actively incorporated into the primary setting due to recent study results [3, 4, 16], discovering a new targeted drug is desperately required to treat recurrent ovarian cancer. Thereafter, disease progression after PARP inhibitors may alter pre-existing PARP1 activity or restore the HR repair pathway [12, 13]. Therefore, new molecular targets such as Chk2 inhibitors are expected to overcome the resistance to PARP inhibitors as well as platinum-resistance for recurrent ovarian cancer.

In conclusion, PHI-101, a selective Chk2 inhibitor, is a promising molecule to overcome the current treatment for platinum-resistant recurrent ovarian cancer, and it is necessary to conduct a study to assess the safety and tolerability of PHI-101.

## Supplementary Information

**Additional file 1.**

**Additional file 2.**

## Data Availability

The datasets used and/or analysed during the current study are available from the corresponding author on reasonable request.

## List of abbreviations

AR: Accumulation ratio
ATM: Ataxia telangiectasia mutated kinase
ATR: Ataxia telangiectasia and Rad3-related kinase
Chk1 and 2: Checkpoint kinase 1 and 2
CTCAE: Common Terminology Criteria for Adverse Events
DDR: DNA damage repair
DLT: Dose-limiting toxicity
ECOG: Eastern Cooperative Oncology Group
e-CRF: Electronic case report form
EOT: End of treatment
HR: Homologous recombination
HRD: Homologous recombination deficiency
ICH-GCP: The International Council for Harmonisation of Technical Requirements for Pharmaceuticals for Human Use-Good Clinical Practice
IP: Investigational product
KGCP: Korea Good Clinical Practice
MTD: Maximum tolerated dose
PARP inhibitor: Poly(ADP-ribose) polymerase inhibitors
PFT: Peak-trough fluctuation
SRC: Safety review committee

## Declarations

### Study status

This study is ongoing and have not completed participant recruitment.
