# Supplementary material for: A phase IA dose-escalation study of PHI-101, a new checkpoint kinase 2 inhibitor, for platinum-resistant recurrent ovarian cancer
Source: BMC Cancer. 2022 Jan 3;22:28. doi: 10.1186/s12885-021-09138-z (PMC8722005; doi:10.1186/s12885-021-09138-z)
Supplement: Supplementary file 1 — Additional file 1. [file 12885_2021_9138_MOESM1_ESM.docx]

Supplementary Table 1. Schedule of activities

| **Cycle/Visit/Period** | **Screen** | **Cycle 1** | | | | **Cycles 2, 3** | | **Cycles n (n≥4)** | **EOT^1^** | **EOS^2^** | **FU Period^3^**  **(EOS+W56)** |
| --- | --- | --- | --- | --- | --- | --- | --- | --- | --- | --- | --- |
|  |  | **Day 1~Day 28** | | | | **Cycle 2: Day 29~Day 56**  **Cycle 3: Day 57~Day 84** | | **Day 85~**  **(28-day cycle)** |  |  |  |
| **Day (D)/week (W)** | **-D28** | **D1**  **(BL)** | **D8** | **D15** | **D22** | **D1** | **D15** | **D1** | **-** | **Q8W** | **Q12W** |
| **Visit window (days)** | **-** | **-** | **±2d** | **±2d** | **±2d** | **±2d** | **±2d** | **±2d** | **±7d** | **±15d** | **±30d** |
| Hospital visit (🕿: phone visit available) | ● | ● | ● | ● | ● | ● | ● | ● | ● | 🕿 | 🕿 |
| Informed consent | ● |  |  |  |  |  |  |  |  |  |  |
| ***Basic subject information*** | | | | | | | | | | | |
| Demographic information | ● |  |  |  |  |  |  |  |  |  |  |
| Information on cancer | ● |  |  |  |  |  |  |  |  |  |  |
| Medical history**^4^** | ● |  |  |  |  |  |  |  |  |  |  |
| Prior/concomitant therapy**^5^** | ● | ● | ● | ● | ● | ● | ● | ● | ● | ● | ● |
| Vital signs**^6^** | ●**^7^** | ● |  | ● |  | ● | ● | ● | ● |  |  |
| Physical measurement**^8^** | Ht/Wt | Wt |  |  |  | Wt |  | Wt | Wt |  |  |
| Physical examination | ●**^7^** | ● |  | ● |  | ● | ● | ● | ● |  |  |
| ECOG PS | ●**^7^** | ● |  | ● |  | ● | ● | ● | ● |  |  |
| Inclusion/exclusion criteria | ● | ● |  |  |  |  |  |  |  |  |  |
| ***Laboratory tests* ^9^** | | | | | | | | | | | |
| Hematology | ●**^7^** | ● | ● | ● | ● | ● | ● | ● | ● |  |  |
| Chemistry | ●**^7^** | ● | ● | ● | ● | ● | ● | ● | ● |  |  |
| Thyroid Function Tests | ●**^7^** | ● |  |  |  |  |  | C6D1, C12D1…(Q6cycles) | ● |  |  |
| Coagulation Profile | ●**^7^** | ● |  |  |  |  |  | C6D1, C12D1…(Q6cycles) | ● |  |  |
| HbA1c | ●**^7^** | ● |  |  |  |  |  | C6D1, C12D1…(Q6cycles) | ● |  |  |
| Urinalysis | ●**^7^** | ● |  |  |  | C2D1, C4D1…(Q2cycles) | | | ● |  |  |
| Viral Serology | ● |  |  |  |  |  |  |  |  |  |  |
| FSH | ● |  |  |  |  |  |  |  |  |  |  |
| CA 125**^10^** | ● | Q8weeks(±1w) from C1D1 based on calendar day (add ★) | | | | | | | ● |  |  |
| Pregnancy test**^11^** | S | S |  |  |  | C2D1, C3D1…(Q1cycle): S(U) | | | S |  |  |
| 12-Lead ECG**^12^** | 3 times**^7^** | 3 times |  |  |  | 1 time (add ★: a total of 3 measurements with 2 additional measurements included) | | | |  |  |
| ECHO or MUGA scan**^13^** | ● | ★ | | | | | | | |  |  |
| ***Tumor response assessment*** | | | | | | | | | | | |
| CT or MRI (abdomen, pelvis, chest + another body part, if necessary)**^14^** | ● | Q8weeks(±1w) from C1D1 based on calendar day (add ★) | | | | | | | ● |  | |
| PK sampling**^15^** |  | 8 times | 1 time | 8 times | 1 time | C2D1/C3D1: 2 times | | C6D1, C9D1…(Q3cycles): 1 time | EOT: 1 time |  |  |
| Administration of PHI-101 and dosing diary completion**^16^** |  | Oral administration until EOT | | | | | | | |  |  |
| DLT observation**^17^** |  | ● | | | |  |  |  |  |  |  |
| Adverse event | ● | ● | ● | ● | ● | ● | ● | ● | ● | ● | ● |
| Checking for new antineoplastic therapy and survival status |  |  |  |  |  |  |  |  |  | ● | ● |

Abbreviations: BL, baseline; CT, computed tomography; ECHO, echocardiography; EOS, end of study; EOT, end of treatment; FU, follow-up, Ht, height; MRI, magnetic resonance imaging; MUGA, multi-gated acquisition blood pool scintigraphy; Q, every; S, serum; U, urine; Wt, weight.

★: If clinically needed

- Each 28 days from the first dose of PHI-101 (C1D1) constitutes 1 cycle. Therefore, D29 which is 28 days after C1D1 will be C2D1, and the same applies to subsequent cycles. Cycles will not be affected by dose interruption (a maximum of 4 weeks) or treatment resumption schedule of PHI-101 and remain unchanged without rescheduling during the study.
- At unscheduled visits (USVs), tests and procedures required based on the judgment of the investigator will be performed.
- The screening visit and baseline (C1D1) visit can occur on the same day.
  1. **End of treatment (EOT)**: If new antineoplastic therapy needs to be initiated in the EOT visit period (EOT±7d), the EOT visit should be made before initiation of new antineoplastic therapy, if possible.

***AE and ADR collection period:*** Until the EOT, all AEs that occur will be collected. After the EOT, only ADRs will be collected (If new antineoplastic therapy is performed before the EOT visit, AEs will be collected until then).

***Follow-up of AEs and ADRs:*** AEs and/or ADRs unresolved at EOT should be followed up, if possible, until resolution (or normalization as judged by the investigator), until EOS, or until further follow-up is determined meaningless.

- 1. **End of study (EOS)**: ① EOT for the last subject or ② at least 12 months after the last subject’s first dose of PHI-101, whichever is earlier

***Every 8 weeks until the EOS (A)***: Information on new antineoplastic therapy and survival status will be followed up via phone visit (hospital visit, if necessary), and if there are any AEs and/or ADRs requiring follow-up, their information may be collected in this period. Also, a tumor response assessment may be performed together in applicable subjects.

***At the EOS (B)***: For items observed (investigated) in (A), all subjects will be investigated collectively.

- 1. **Follow-up (FU) period**: for 1 year from the EOS.

***Every 12 weeks until the end of the FU period (C)***: The same items observed (investigated) in (A) of the EOS will be investigated via phone visit (hospital visit, if necessary).

***At the end of the FU period (D)***: For items observed (investigated) in (A) of the EOS, all subjects will be investigated collectively.

- 1. **Medical history**: Medical history within 24 weeks prior to Visit 1 will be investigated.
  2. **Prior/concomitant therapy**: For prior therapy, medication history (treatment history) within 24 weeks prior to Visit 1 will be investigated.
  3. **Vital signs**: Blood pressure (systolic/diastolic), pulse, respiratory rate, and body temperature will be measured after resting in a sitting position for 5 minutes or longer.
  4. **Replacement of tests scheduled for C1D1**: If screening is performed within 7 days from baseline (C1D1), tests scheduled for C1D1 may be replaced with test results at screening.
  5. **Physical measurement**: Height and body weight will be measured. Height will be measured only at screening.
  6. **Laboratory tests**: For laboratory tests, a re-test may be performed during the screening period, and the final test result will be used to determine if the subject meets the inclusion/exclusion criteria. For chemistry (blood glucose), subjects should fast for at least 8 hours before blood sampling.
  7. **CA 125**: will be measured simultaneously with the radiographic tumor response assessment.
  8. **Pregnancy test**: For women of childbearing potential, a serum hCG test will be performed at screening, C1D1, and the EOT. At CnD1 (n≥2) and when necessary, a urine-hCG (and/or serum hCG) test will be performed.
  9. **12-lead ECG**: will be performed 3 times at screening and C1D1 and only once at subsequent cycles. However, if QT interval prolongation is demonstrated in the ECG, additional ECG will be performed twice in a short period time (immediately) to determine discontinuation of the subject from the study based on the mean QTc interval of 3 ECG measurements.
  10. **ECHO or MUGA scan**: will be performed at screening and as clinically indicated by the investigator. Test results may be replaced with available results obtained within 12 weeks prior to informed consent.
  11. **Radiographic tumor response assessment**:

• It will be performed at screening, if no CT scan result (abdomen, pelvis, chest, etc.) obtained within 28 days prior to baseline (C1D1) is available.

• For subjects with current or history of hypersensitivity to CT scan contrast, it may be replaced with MRI at the judgment of the investigator.

• The same imaging should be performed at every assessment time point and baseline (C1D1).

• The tumor response assessment will be scheduled following calendar days, and it must not be adjusted due to delay in the start of a cycle, etc. For CT scan (or MRI), subjects may make a visit according to their hospital visit schedule within ±1 week from the scheduled tumor response assessment.

• An investigator’s tumor response assessment must be performed within 7 days from imaging.

• A tumor response assessment will be performed every 8 weeks (±1 week) from C1D1 and at the EOT visit. If CT scan (or MRI) result obtained within 4 weeks prior to the EOT visit is available, the scan does not need to be repeated at the EOT. Also, a tumor response assessment will be performed as clinically indicated by the investigator (e.g., when progressive disease [PD] is suspected). If the IP is discontinued due to PD, additional tumor response assessments do not need to be performed after PD d.

• For subjects with no confirmed PD until the EOT visit, a tumor response assessment will be performed every 8 weeks (±1 week) until the ‘EOS, confirmation of PD, initiation of new antineoplastic therapy, consent withdrawal or death’, whichever is earliest.

- 1. **PK sampling**: PK sampling timepoints scheduled for each cycle. Samples for PK analysis may be additionally collected as needed at the judgment of the investigator.
  2. **Administration of PHI-101 and dosing diary completion**: Subjects will be orally administered PHI-101 (2 to 12 tablets/day) at a predetermined dose for the assigned cohort once daily each day for 28 days (1 cycle). Subjects will record dosing details (dosing day, dosing time, dose, etc.) of PHI-101 in the dosing diary and bring it along with dispensed PHI-101 at every visit so that the investigator can check treatment compliance and reasons for dose interruption. The investigator will distribute a new dosing diary to subjects every cycle and collect the completed dosing diary.
  3. **DLT assessment**: DLT will be assessed during the first 1 cycle (28 days) after administration of PHI-101.

Supplementary Table 2 Possible toxicities shown in preclinical study results

| Category | Specific events |
| --- | --- |
| Gastrointestinal | Vomiting on the day of administration, abnormal stools, salivation, decreased appetite (verified by decrease in food consumption) |
| Body as a whole: | Decreased activity, partial eyelid closure, tachypnea, somnolence |
| Liver | Increased ALP, AST, ALT, GGT, and total bilirubin |
| Hematology | Abnormal RBC count, hematocrit, hemoglobin, mean corpuscular volume, platelet count, and WBC count |
| Chemistry | Increased blood urea nitrogen and creatinine |
| Other laboratory results | Increased globulin and carbohydrate, decreased albumin, albumin/globulin ratio, and glucose |
| Gross anatomy | Decrease in size of thymus and spleen, intestinal discoloration, ovary discoloration in females, increase in size and volume of liver |
| Microscopy | Decreased lymphoid cellularity in thymus, spleen, lymph nodes, and Peyer’s patches |

Abbreviations: ALP, alkaline phosphatase; AST, aspartate transaminase; ALT, alanine aminotransferase; GGT, γ-glutamyl transferase; RBC, red blood cell; WBC, white blood cell.
