# Supplementary material for: A phase IA dose-escalation study of PHI-101, a new checkpoint kinase 2 inhibitor, for platinum-resistant recurrent ovarian cancer
Source: BMC Cancer. 2022 Jan 3;22:28. doi: 10.1186/s12885-021-09138-z (PMC8722005; doi:10.1186/s12885-021-09138-z)
Supplement: Supplementary file 2 — Additional file 2. [file 12885_2021_9138_MOESM2_ESM.pdf]

Electronic Case Report Form

PROTOCOL	PHI-101-002
STUDY TITLE	백금 저항성/불응성 난소암 환자 대상 PHI-101의 안전성, 내약성 및 약동학적 특성을 평가하기 위한 공개, 용량결정, 제1상 임상시험<br>A Phase I, Open-label, Dose-finding Study to Assess the Safety, Tolerability and Pharmacokinetics of PHI-101 in Patients with Platinum-Resistance/Refractory Ovarian Cancer

Site Name	-
CRF Version	2.1
CRF Effective	2021-07-06
Screening Number	-

	등록	Screen	C1D1	C1D8	C1D15	C1D22	C2D1	C2D15	C3D1	C3D15
등록	☐									
방문일		☐	☐	☐	☐	☐	☐	☐	☐	☐
인구학적 정보		☐								
암 관련 정보		☐								
병력		☐								
활력징후, 신체계측		☐	☐		☐		☐	☐	☐	☐
신체검진		☐	☐		☐		☐	☐	☐	☐
ECOG PS		☐	☐		☐		☐	☐	☐	☐
임신검사		☐	☐				☐		☐	
실험실 검사		☐	☐	☐	☐	☐	☐	☐	☐	☐
12-Lead ECG		☐	☐				☐	☐	☐	☐
ECHO 또는 MUGA scan		☐	☐	☐	☐	☐	☐	☐	☐	☐
선정/제외 기준		☐	☐							
PK 채혈			☐	☐	☐	☐	☐		☐	
PHI-101 투여										
임상시험 투여종료										
DLT 관찰										
CA 125 검사										
종양평가										
이상반응										

	등록	Screen	C1D1	C1D8	C1D15	C1D22	C2D1	C2D15	C3D1	C3D15
선행/병용 약물										
선행/병용 요법										
새로운 항암 요법 확인										
생존여부 확인										
임상시험 종료										
시험책임자 서명										

	C4D1	EOT	USV1	FU1-1	EOS	All1	FU2-1	All2
등록								
방문일	☐	☐	☐	☐	☐		☐	
인구학적 정보								
암 관련 정보								
병력								
활력징후, 신체계측	☐	☐	☐					
신체검진	☐	☐	☐					
ECOG PS	☐	☐	☐					
임신검사	☐	☐	☐					
실험실 검사	☐	☐	☐					
12-Lead ECG	☐	☐	☐					
ECHO 또는 MUGA scan	☐	☐	☐					
선정/제외 기준								
PK 채혈	☐	☐						
PHI-101 투여						☐		
임상시험 투여종료		☐						
DLT 관찰						☐		
CA 125 검사								☐
종양평가								☐
이상반응								☐
선행/병용 약물								☐

	C4D1	EOT	USV1	FU1-1	EOS	All1	FU2-1	All2
선행/병용 요법								☐
새로운 항암 요법 확인								☐
생존여부 확인								☐
임상시험 종료						☐		☐
시험책임자 서명						☐		☐

등록	
시험대상자 동의서 서명일을 기재하십시오.	
동의서 서명일	______
재스크리닝 대상자입니까?	○ 예<br>○ 아니오
이전 스크리닝 번호	____
용량수준(Cohort)	○ 1<br>○ 2<br>○ 3<br>○ 4<br>○ 5<br>○ 6
DLT 평가불능 대상자를 대체합니까?	○ 예<br>○ 아니오
스크리닝 번호	______

방문일	
방문일	______

선행/병용약물 확인
Screen방문 기준 24주 이내 투여되었거나 현재 투여중인 약물이 있는 경우 선행/병용약물 페이지에 기록하여 주십시오.

이상반응 확인
동의서 취득 이후부터 발생한 이상반응이 있을 경우 이상반응 페이지에 기록하여 주십시오.

인구학적 정보	
생년월	____
연령[자동계산]	만 _ 세
성별	○ 남성<br>○ 여성
가임 여부	○ 예<br>○ 아니오

비가임 여성은 아래의 조건 중 1개 이상 충족하는 경우로 정의한다.
- 12개월 동안 자연적인 무월경
- 혈중 FSH 농도가 40 mIU/mL를 초과하면서 6개월 동안의 자연적인 무월경
- 외과적 불임수술 자궁적출술, 난소절제술, 난관결찰술 등을 받은 경우

난소암 기초정보

암 진단 여부	○ 예<br>○ 아니오
난소암 종류(원발장기/부위)	☐ 난소암<br>☐ 난관암<br>☐ 원발성 복막암
난소암 최초 진단일	______
유병기간[자동계산]	__ 일
등록시점의 병기	___▼

등록시점의 병기	Stage I , Stage II , Stage III , Stage IV , Other
등록시점의 병기_Other	______________________________<br>______________________________<br>______________________________

전이 여부	○ 예<br>○ 아니오
전이 부위	☐ 뼈<br>☐ 뇌<br>☐ 간<br>☐ 폐<br>☐ 림프절<br>☐ 기타
전이 부위, 기타 상세	______________________________<br>______________________________<br>______________________________

백금기반 치료 유형	○ 불응성<br>○ 저항성

* 불응성: 백금기반 항암요법 진행중, 질병이 진행함
* 저항성: 백금기반 항암요법 완료 수, 6개월(24주) 이내에 질병이 진행함

조직학적/세포학적 유형

상피성암	○ 예<br>○ 아니오
흔하지 않는 난소 조직병리(LCOH)	○ 예<br>○ 아니오

항암화학요법

None	☐ None

* 항암 화학요법 (regimen)의 구체적인 내용을 아래에 기록하십시오. 약물명은 regimen 단위로 기재합니다.
* 해당 요법의 각각의 약물명과 투여 경로를 기록하고, 총 투여 주기수, 시작일, 종료일, 목적, 변경사유는 항암요법 (regimen)을 기준으로 작성합니다.

No	약물명	경로	총 투여 주기	시작일	종료일	목적	변경사유	최상의 전반적 반응
1	____<br>____<br>____	____<br>____<br>____	____	____	____	___▼	___▼	___▼

목적	Neoadjuvant , Adjuvant , Palliative , Unknown
변경사유	Resistance , Refractory , Intolerability , Cumulative dose , Other
변경사유_Other	____<br>____<br>____
최상의 전반적 반응	CR , PR , SD , PD , NE

수술요법

None	☐ None			
No	수술명	수술일	부위	목적
1	______ ______ ______	______	______ ______ ______	○ Curative ○ Palliative

방사선요법

None	☐ None				
No	방사선 조사부위	시행일	종료일	총 방사선량(Gy)	목적
1	___▼	______	______	______ ______ ______	___▼

방사선 조사부위	난소 , 난관 , 위 , 대장 , 직장 , 신장 , 전립선 , 방광 , 췌장 , 간 , 쓸개 및/또는 담도 , 유방 , 자궁경부 , 갑상선 , 폐(비소세포) , 폐(소세포) , 근육/뼈 및/또는 결합조직 , 악성 복수 , 악성 흉수 , 기타
방사선 조사부위_기타	______ ______ ______
목적	Neoadjuvant , Adjuvant , Palliative , Unknown

면역요법/호르몬요법

None	☐ None						
No	약물명	경로	투여일	종료일	목적	변경사유	최상의 전반적 반응
1	______ ______ ______	______ ______ ______	______	______	___▼	___▼	___▼

목적	Neoadjuvant , Adjuvant , Palliative , Unknown

변경사유	Refractory , Intolerability , Cumulative dose , Other
변경사유_Other	______________________________<br>______________________________<br>______________________________
최상의 전반적 반응	CR , PR , SD , PD , NE

HRD 관련 유전자

None	☐ None

BRCA1, BRCA2, FANC, ATM, MRE11A, RAD51, RAD54, ATR, DSS1, RPA1, NBS1, CHEK1, CHEK2 등 해당하는 항목을 기재 후 검사일 및 wild type/mutation 여부를 기재합니다.

No	항목	검사일	wild type/mutation 여부
1	______________________________<br>______________________________<br>______________________________	_____	___▼

wild type/mutation 여부	wild type , mutation , NA

병력	
None	☐ None

* 대상자의 동의 취득 전까지 관찰된 임상적으로 유의한 의학적 상태 또는 비정상은 병력으로 간주합니다.
* Screen 방문 기준 지난 24주 이내의 병력(외과적 수술/시술력 포함)을 조사합니다.
 단, 선정/제외 기준과 관련된 병력의 경우, 계획서에 따라 검토되어야 하는 기간에 준하여 모두 조사하여야 한다.
* 외과적 수술(시술 포함)의 경우 '수술명'과 '수술을 하게 된 원인 질환명'을 각각 기재합니다.

No	의학적 상태/진단명	진단일/수술일	방문 당시 지속여부	종료일	Comment
1	________<br>________<br>________	____	○ 예<br>○ 아니오<br>○ 해당없음(시술 또는 수술)	____	________<br>________<br>________

활력징후	
ND	☐ ND
미실시인 경우 사유	____<br>____<br>____
5분 이상 안정을 취한 후 앉은 자세(좌위)에서 측정합니다.	
측정일	______
수축기 혈압	__ mmHg
이완기 혈압	__ mmHg
맥박	__ 회/분
호흡수	__ 회/분
체온	_._ ℃
임상적으로 유의한 비정상 소견이 있습니까?	○ 예<br>○ 아니오
대상자의 동의 취득 전까지 관찰된 임상적으로 유의한 비정상 소견이 있을 경우, 비정상 소견은 '병력'에 기록하십시오.	

신체계측	
ND	☐ ND
미실시인 경우 사유	____<br>____<br>____
측정일	______
신장	__ cm

체중	__. kg

신체검진

ND	☐ ND
미실시인 경우 사유	______
검진일	______
각 신체기관 검진 결과 비정상 소견이 있습니까?	○ 예 → 아래 표에 신체기관 및 상세내용을 기재하여 주십시오.<br>○ 아니오

대상자의 동의 취득 전까지 관찰된 임상적으로 유의한 비정상 소견이 있을 경우, 비정상 소견은 '병력'에 기록하십시오.

No	신체기관	임상적 유의성
1	___▼	○ 예 ○ 아니오

신체기관	외관 , 피부 , 두/경부 , 흉부/폐 , 심장 , 복부 , 비뇨/생식계 , 사지 , 근골격계 , 신경계 , 림프절 , 기타
신체기관_기타	______

ECOG PS	
ND	☐ ND
미실시인 경우 사유	___<br>___<br>___
평가일	___
ECOG PS	○ Grade 0<br>○ Grade 1<br>○ Grade 2<br>○ Grade 3<br>○ Grade 4<br>○ Grade 5

임신검사	
ND	☐ ND
미실시인 경우 사유	______________________<br>______________________<br>______________________
검사일	______
검사 방법	○ urine-hCG<br>○ serum-hCG
검사결과	○ 양성<br>○ 음성
serum-hCG 검사 방법으로 실시합니다.	

실험실 검사	
ND	☐ ND
미실시인 경우 사유	______<br>______<br>______
최소 8시간 금식 여부	○ 예<br>○ 아니오
검체 채취일	______

* 동의서 취득 전 검사 결과가 'Clinical Significance' 일 경우 비정상 소견은 '병력'에 기록하십시오.
* 검사 항목별 단위는 "정상 참고 범위"와 함께 제공되므로 아래 결과에 기록하지 않습니다.

Hematology			
ND	☐ ND		
검사항목	결과	정상 여부	임상적 유의성
WBC	______	○ 정상 ○ 비정상	○ 예 ○ 아니오
RBC	______	○ 정상 ○ 비정상	○ 예 ○ 아니오
Hemoglobin	______	○ 정상 ○ 비정상	○ 예 ○ 아니오
Hematocrit	______	○ 정상 ○ 비정상	○ 예 ○ 아니오
Platelet	______	○ 정상 ○ 비정상	○ 예 ○ 아니오
MCV	______	○ 정상 ○ 비정상	○ 예 ○ 아니오
MCH	______	○ 정상 ○ 비정상	○ 예 ○ 아니오
MCHC	______	○ 정상 ○ 비정상	○ 예 ○ 아니오
Neutrophils	______	○ 정상 ○ 비정상	○ 예 ○ 아니오

검사항목	결과	정상 여부	임상적 유의성
Lymphocytes	______	○ 정상 ○ 비정상	○ 예 ○ 아니오
Monocytes	______	○ 정상 ○ 비정상	○ 예 ○ 아니오
Eosinophils	______	○ 정상 ○ 비정상	○ 예 ○ 아니오
Basophils	______	○ 정상 ○ 비정상	○ 예 ○ 아니오
ANC	______	○ 정상 ○ 비정상	○ 예 ○ 아니오

Chemistry			
ND	☐ ND		
검사항목	결과	정상 여부	임상적 유의성
BUN	______	○ 정상 ○ 비정상	○ 예 ○ 아니오
Creatinine	______	○ 정상 ○ 비정상	○ 예 ○ 아니오
Total bilirubin	______	○ 정상 ○ 비정상	○ 예 ○ 아니오
AST	______	○ 정상 ○ 비정상	○ 예 ○ 아니오
ALT	______	○ 정상 ○ 비정상	○ 예 ○ 아니오
ALP	______	○ 정상 ○ 비정상	○ 예 ○ 아니오
ɣGT	______	○ 정상 ○ 비정상	○ 예 ○ 아니오
LDH	______	○ 정상 ○ 비정상	○ 예 ○ 아니오
Total protein	______	○ 정상 ○ 비정상	○ 예 ○ 아니오
Albumin	______	○ 정상 ○ 비정상	○ 예 ○ 아니오
Globulin[자동계산]	______	○ 정상 ○ 비정상	○ 예 ○ 아니오

검사항목	결과	정상 여부	임상적 유의성
Ca	______	○ 정상 ○ 비정상	○ 예 ○ 아니오
Mg	______	○ 정상 ○ 비정상	○ 예 ○ 아니오
Na	______	○ 정상 ○ 비정상	○ 예 ○ 아니오
K	______	○ 정상 ○ 비정상	○ 예 ○ 아니오
Cl	______	○ 정상 ○ 비정상	○ 예 ○ 아니오
P	______	○ 정상 ○ 비정상	○ 예 ○ 아니오
Total cholesterol	______	○ 정상 ○ 비정상	○ 예 ○ 아니오
Triglyceride	______	○ 정상 ○ 비정상	○ 예 ○ 아니오
CPK	______	○ 정상 ○ 비정상	○ 예 ○ 아니오
Glucose	______	○ 정상 ○ 비정상	○ 예 ○ 아니오
CrCl[자동계산]	______		

Urinalysis

ND	☐ ND		
검사항목	결과	정상 여부	임상적 유의성
Specific gravity	______	○ 정상 ○ 비정상	○ 예 ○ 아니오
pH	______	○ 정상 ○ 비정상	○ 예 ○ 아니오
Bilirubin[U]	______	○ 정상 ○ 비정상	○ 예 ○ 아니오
Urobilinogen[U]	______	○ 정상 ○ 비정상	○ 예 ○ 아니오
Blood[U]	______	○ 정상 ○ 비정상	○ 예 ○ 아니오

검사항목	결과	정상 여부	임상적 유의성
Glucose[U]	______	○ 정상 ○ 비정상	○ 예 ○ 아니오
Protein[U] (Alb[U])	______	○ 정상 ○ 비정상	○ 예 ○ 아니오
Ketones[U]	______	○ 정상 ○ 비정상	○ 예 ○ 아니오
Leukocytes[U]	______	○ 정상 ○ 비정상	○ 예 ○ 아니오

Thyroid Function Tests

ND	☐ ND		
검사항목	결과	정상 여부	임상적 유의성
TSH	______	○ 정상 ○ 비정상	○ 예 ○ 아니오
Free T4	______	○ 정상 ○ 비정상	○ 예 ○ 아니오

Tests for HbA1c

ND	☐ ND		
검사항목	결과	정상 여부	임상적 유의성
HbA1c	______	○ 정상 ○ 비정상	○ 예 ○ 아니오

Coagulation Profile

ND	☐ ND		
검사항목	결과	정상 여부	임상적 유의성
PT INR	______	○ 정상 ○ 비정상	○ 예 ○ 아니오
aPTT	______	○ 정상 ○ 비정상	○ 예 ○ 아니오
Fibrinogen	______	○ 정상 ○ 비정상	○ 예 ○ 아니오

검사항목	결과	정상 여부	임상적 유의성
D-dimer	______	○ 정상 ○ 비정상	○ 예 ○ 아니오

Viral Serology	
ND	☐ ND
검사항목	결과
HBsAg	○ ND ○ Positive ○ Negative
HBV DNA	○ ND ○ Positive ○ Negative
anti-HCV	○ ND ○ Positive ○ Negative
HCV RNA	○ ND ○ Positive ○ Negative

FSH			
ND	☐ ND		
검사항목	결과	정상 여부	임상적 유의성
FSH	______	○ 정상 ○ 비정상	○ 예 ○ 아니오

12-Lead ECG

ND	☐ ND
미실시인 경우 사유	______<br>______<br>______
검사일	______
측정횟수	○ 1회<br>○ 3회
BBB 여부	○ 예<br>○ 아니오

No.	Heart Rate	PR Interval	QRS Duration	QT Interval	QTcB [자동계산]	QTcF [자동계산]
1차	__ bpm	__ msec	__ msec	__ msec	__._	__._
2차	__ bpm	__ msec	__ msec	__ msec	__._	__._
3차	__ bpm	__ msec	__ msec	__ msec	__._	__._

QTcB 평균[자동계산]	__._
QTcF 평균[자동계산]	__._
임상적으로 유의한 비정상 소견이 있습니까?	○ 예<br>○ 아니오

* 대상자의 동의 취득 전까지 관찰된 임상적으로 유의한 비정상 소견이 있을 경우, 비정상 소견은 '병력'에 기록하십시오.
* Screen, C1D1 시점에서는 3회 측정하고, 이후 주기에서는 1회만 수행합니다.
(단, ECG를 통해 QT 간격 연장이 입증된 경우, 단기간(즉시)에 추가로 2개의 ECG를 수행하여 최종 3개 ECG의 평균 QTc interval을 확인합니다.)

ECHO 또는 MUGA scan	
ND	☐ ND
미실시인 경우 사유	________<br>________<br>________
검사일	______
검사 방법	○ ECHO<br>○ MUGA scan
좌심실구출률(LVEF)	__._ %
임상적으로 유의한 비정상 소견이 있습니까?	○ 예<br>○ 아니오
* 스크리닝 전 12주 이내에 실시된 검사결과가 있는 경우, 해당 검사결과로 대체할 수 있습니다.<br>* 대상자의 동의 취득 전까지 관찰된 임상적으로 유의한 비정상 소견이 있을 경우, 비정상 소견은 '병력'에 기록하십시오.	

시험대상자 적합성	
상기 시험대상자는 선정/제외 기준을 만족하고, 임상시험 참여에 적합합니까?	○ 예<br>○ 아니오
탈락사유	○ 선정/제외기준 부적합 → Specify below<br>○ 시험대상자 동의철회<br>○ 기타 → Specify below
탈락사유 상세	______<br>______<br>______

선정기준
대상자는 다음의 모든 기준에 적격하여야 본 임상에 참여할 수 있다.
1. 서면동의일 기준으로 만 19세 이상의 여성
2. 임신(임신가능성, 임신계획), 수유 여부<br>① 비가임 여성, 임신 또는 수유 중이 아닌 여성, 임상시험 기간 동안 임신계획이 없는 여성<br>② 가임 여성인 경우, 스크리닝 기간 및 PHI-101 투여 직전의 임신반응검사에서 음성으로 확인되고, 본 계획서에서 요구하는 효과적인 피임법을 PHI-101의 마지막 투여 후 6개월(24주)간 준수하기로 동의한 경우
3. 대상질환<br>① 조직학적(histologically) 또는 세포학적(cytologically)으로 확인된 난소암, 난관암 또는 원발성 복막암<br>② 국소조직학적 소견(local histopathological findings)으로 진단된 상피성암(epithelial malignant tumors) [WHO Histological Classification, 2014]<br>: 단, 흔하지 않는 난소 조직병리(LCOH)로 분류되는, 저등급장액성난소암(LGSC), 점액성난소암(mucinous carcinoma of the ovary), 암육종(악성혼합뮐러종양[MMMT]), 경계성상피성난소종양(저악성암종[LAP]) 제외 [NCCN Guideline version 2.2019]<br>③ 백금불응성암* 또는 백금저항성암**<br>*백금기반 항암요법 진행중, 질병이 진행함, **백금기반 항암요법 완료 후, 6개월(24주) 이내에 질병이 진행함<br>④ 현재 임상적 이익이 있는 것으로 알려진 표준치료법에 대해 불응하거나, 받을 수 없거나, 거부한 자로 수술이 불가한 경우<br>⑤ [RECIST version 1.1]에 부합하는, 최소 한 개 이상의 측정 가능한 병변 또는 측정 불가하나 평가 가능한 병변을 가진 경우
4. 예상 생존기간 ≥ 12주
5. [ECOG PS] ≤ 1

6. 다음의 실험실적 검사를 통해 적절한 간, 신장, 혈액학적 기능이 확인된 자(스크리닝 기간 동안 재검 가능하다)
① ANC ≥ 1,500/mm³ (단, 베이스라인 이전 2주 이내에 G-CSF 투여 없이)
② Hb ≥ 10.0 g/dL (단, 베이스라인 이전 2주 이내에 수혈 없이)
③ Platelet count ≥ 75,000/mm³ (단, 베이스라인 이전 2주 이내에 수혈 없이)
④ Total bilirubin ≤ 1.5 x ULN
⑤ AST and ALT ≤ 3.0 x ULN* (단, 간전이 또는 간세포암인 경우 ≤ 5 x ULN)
⑥ Serum creatinine ≤ 1.5 x ULN (or CrCl ≥ 60 mL/min (by Cockcroft-Gault equation)

7. 이전 항암요법 및 치료
① 이전 항암화학요법 ≤ 5 회
② 이전 항암요법(수술, 약물, 방사선치료 등)*들의 가역적인 부작용들(reversible side effects)로부터 [CTCAE version 5.0] grade 1 또는 그보다 양호한 상태로 회복된 경우
*대수술(major surgery), 항암요법(antineoplastic therapy) 또는 실험적인 치료(experimental therapy), 조혈부위의 직접적인 방사선요법은 베이스라인 이전4주 이내에 적용(투여)받지 않았어야 하며, nitrosoureas 또는 mitomycin-C는 베이스라인 이전 6주 이내에 투여 받지 않았어야 함.

8. 본 임상시험에 대해 충분한 설명을 들은 후 자의로 참여를 결정하고 서면 동의한 자

9. 본 임상시험의 절차 및 제한 사항 등을 이해하고, 임상시험 참여기간 동안 이를 준수할 의사가 있는 자

제외기준

대상자는 다음 조건의 어느 하나라도 해당되는 경우 본 임상시험에 참여할 수 없다.

1. PHI-101의 주성분 또는 부형제에 대해 알려진 또는 의심되는 과민반응 또는 불내성(intolerance)이 있는 경우

2. 시험자가 판단하였을 때 본 임상시험 참여가 부적합 또는 불가능한 환자

3. 다음과 같은 심장기능 부전 또는 심혈관계 질환(단, 이에 국한되지 않는다)
① 베이스라인 이전12주 이내에 발생한 심근허혈의 증거 또는 심근경색
② [NYHA Functional Classification] ≥ II
③ ECHO 또는 MUGA scan을 통해 확인된 LVEF ＜ 50%
④ 임상적으로 유의한, 최적의 적절한 요법에 의해 조절되지 않는 심부정맥
⑤ Corrected QT (QTc)* interval ＞ 450 msec (남/녀 동일), Long QT 증후군(또는 이의 가족력)
*Fridericia 공식에 의해 보정된 QT 간격(QTcF)을 이용한다. 단, 각차단(bundle branch block)이 있을 시 Bazett 공식으로 계산한다(QTcB).

4. 다음과 같은 약물 복용 또는 흡수에 영향을 주는 위장관 질환(단, 이에 국한되지 않는다)
① 연하곤란
② 장관마비, 장폐색
③ 약물 흡수에 임상적으로 유의한 영향을 주는 위장관 수술: 위절제술, 소장루, 광범위한 소장절제술 등(단, 단순 문합술은 제외된다)
④ 위장관계 전체 또는 소장을 침범하는 자가면역 및 염증성 질환: 셀리악병(coeliac disease), 장의 이식편대숙주질환, 베체트신드롬, 위장관을 침범한 경피증(scleroderma), 크론병(Crohn's disease), 궤양성 대장염(ulcerative colitis) 등

5. 다음과 같은 폐질환(단, 이에 국한되지 않는다)
① 새로이 시작된 또는 진행성의 호흡곤란, 기침 및 발열 증상이 있는 경우
② 간질성 폐질환 진단 대기 중인 환자 또는 간질성 폐렴
③ 폐섬유증

6. 림프종(lymphoma)을 포함한 혈액암(hematologic malignancy)
7. 다음과 같은 전이 환자<br>① 중추신경계전이 또는 뇌전이<br>② 골전이
8. 다음과 같은 감염질환(단, 이에 국한되지 않는다)<br>① 임상시험기간 중 안전성 및 유효성 평가에 영향을 줄 수 있는 항생제, 항바이러스제 등의 투여를 요하는 중증의 감염질환<br>② 최적의 적절한 요법에 의해 조절되지 않는 활성(현성) 감염질환으로 시험자가 판단하는 경우
9. 알려진 인체 면역결핍 바이러스(HIV) 양성
10. 활동성B 형 간염* 또는 활동성 C 형 간염**<br>*HBsAg 양성이면서 HBV DNA 가 검출됨. **Anti-HCV 양성이면서 HCV RNA가 (정성적으로)검출됨
11. 서면동의일 기준 12주 이내의 의도하지 않은(unintentional) 체중 감소 〉 10%인 경우
12. 서면동의일 기준 1년(52주) 이내의 알코올 또는 다른 약물의 남용 이력
13. 임상시험 참여기간 동안 임상시험용 의약품 외의 다른 항암요법*이 필요한 경우(단, 기관지 폐쇄, 피부 병변 등의 완화를 목적으로 하는 국소 방사선 조사(point radiation)는 허용된다).<br>*수술, 방사선(화학)요법, 항암화학요법, 표적요법제(저분자의약품, 단일클론항체), 면역항암제(생물학적제제), 또는 호르몬요법 등.
14. 다른 임상시험용 의약품이나 임상시험용 의료기기를 서면동의일 기준 2주 이내, 또는 이의 반감기의 5배에 해당하는 기간 이내(이중 더 짧은 기간)에 투여(적용) 받은 경우
15. 임상시험용 의약품의 동태(체내에서의 대사 및 배설 등) 또는 유효성과 안전성 평가에 유의한 영향을 미치는 약물(비처방약, 허브 또는 동종요법 등 포함)을 스크리닝 이전 2주 이내 투여 중인 것으로 시험자가 판단하는 경우

방문일	
방문일	______
Screen & Cycle1 D1(C1D1) 방문 동시 진행	☐

선행/병용약물 확인
지난 방문과 비교하였을 때, 새롭게 투여되었거나 투여 용량이 변경된 병용약물이 있는 경우 선행/병용약물 페이지에 기록하여 주십시오.

이상반응 확인
지난 방문과 비교하였을 때, 새롭게 발생한 이상반응이 있을 경우 이상반응 페이지에 기록하여 주십시오.<br>(만약, EOT방문 전 새로운 항암요법이 수행되는 경우에는 해당 시점까지만 이상반응을 수집합니다.)

활력징후	
ND	☐ ND
미실시인 경우 사유	______<br>______<br>______
5분 이상 안정을 취한 후 앉은 자세(좌위)에서 측정합니다.	
측정일	______
수축기 혈압	__ mmHg
이완기 혈압	__ mmHg
맥박	__ 회/분
호흡수	__ 회/분
체온	__.__ ℃
임상적으로 유의한 비정상 소견이 있습니까?	○ 예<br>○ 아니오
동의서 취득 이후 임상적으로 유의한 비정상 소견이 있을 경우, 비정상 소견은 '이상반응'에 기록하십시오.	

신체계측	
ND	☐ ND
미실시인 경우 사유	______<br>______<br>______
측정일	______
체중	__.__ kg

신체검진

ND	☐ ND
미실시인 경우 사유	______________<br>______________<br>______________
검진일	______
지난 방문과 비교하였을 때, 각 신체기관 검진 결과 새로운 비정상 소견이 있습니까?	○ 예 → 아래 표에 신체기관 및 상세내용을 기재하여 주십시오.<br>○ 아니오

동의서 취득 이후 임상적으로 유의한 비정상 소견이 있을 경우, 비정상 소견은 '이상반응'에 기록하십시오.

No	신체기관	임상적 유의성
1	___▼	○ 예 ○ 아니오

신체기관	외관 , 피부 , 두/경부 , 흉부/폐 , 심장 , 복부 , 비뇨/생식계 , 사지 , 근골격계 , 신경계 , 림프절 , 기타
신체기관_기타	______________<br>______________<br>______________

ECOG PS	
ND	☐ ND
미실시인 경우 사유	______<br>______<br>______
평가일	______
ECOG PS	○ Grade 0<br>○ Grade 1<br>○ Grade 2<br>○ Grade 3<br>○ Grade 4<br>○ Grade 5

임신검사	
ND	☐ ND
미실시인 경우 사유	______________________<br>______________________<br>______________________
검사일	______
검사 방법	○ urine-hCG<br>○ serum-hCG
검사결과	○ 양성<br>○ 음성
serum-hCG 검사 방법으로 실시합니다.	

실험실 검사	
ND	☐ ND
미실시인 경우 사유	____________<br>____________<br>____________
최소 8시간 금식 여부	○ 예<br>○ 아니오
검체 채취일	______

* 베이스라인(C1D1) 방문이 Screen 방문으로부터 7일 이내일 경우, Screen검사 결과로 대체 할 수 있습니다.
* 동의서 취득 이후 검사 결과가 'Clinical Significance' 일 경우 비정상 소견은 '이상반응'에 기록하십시오.
* 검사 항목별 단위는 "정상 참고 범위"와 함께 제공되므로 아래 결과에 기록하지 않습니다.

Hematology			
ND	☐ ND		
검사항목	결과	정상 여부	임상적 유의성
WBC	______	○ 정상 ○ 비정상	○ 예 ○ 아니오
RBC	______	○ 정상 ○ 비정상	○ 예 ○ 아니오
Hemoglobin	______	○ 정상 ○ 비정상	○ 예 ○ 아니오
Hematocrit	______	○ 정상 ○ 비정상	○ 예 ○ 아니오
Platelet	______	○ 정상 ○ 비정상	○ 예 ○ 아니오
MCV	______	○ 정상 ○ 비정상	○ 예 ○ 아니오
MCH	______	○ 정상 ○ 비정상	○ 예 ○ 아니오
MCHC	______	○ 정상 ○ 비정상	○ 예 ○ 아니오
Neutrophils	______	○ 정상 ○ 비정상	○ 예 ○ 아니오

검사항목	결과	정상 여부	임상적 유의성
Lymphocytes	______	○ 정상 ○ 비정상	○ 예 ○ 아니오
Monocytes	______	○ 정상 ○ 비정상	○ 예 ○ 아니오
Eosinophils	______	○ 정상 ○ 비정상	○ 예 ○ 아니오
Basophils	______	○ 정상 ○ 비정상	○ 예 ○ 아니오
ANC	______	○ 정상 ○ 비정상	○ 예 ○ 아니오

Chemistry			
ND	☐ ND		
검사항목	결과	정상 여부	임상적 유의성
BUN	______	○ 정상 ○ 비정상	○ 예 ○ 아니오
Creatinine	______	○ 정상 ○ 비정상	○ 예 ○ 아니오
Total bilirubin	______	○ 정상 ○ 비정상	○ 예 ○ 아니오
AST	______	○ 정상 ○ 비정상	○ 예 ○ 아니오
ALT	______	○ 정상 ○ 비정상	○ 예 ○ 아니오
ALP	______	○ 정상 ○ 비정상	○ 예 ○ 아니오
ɣGT	______	○ 정상 ○ 비정상	○ 예 ○ 아니오
LDH	______	○ 정상 ○ 비정상	○ 예 ○ 아니오
Total protein	______	○ 정상 ○ 비정상	○ 예 ○ 아니오
Albumin	______	○ 정상 ○ 비정상	○ 예 ○ 아니오
Globulin[자동계산]	______	○ 정상 ○ 비정상	○ 예 ○ 아니오

검사항목	결과	정상 여부	임상적 유의성
Ca	______	○ 정상 ○ 비정상	○ 예 ○ 아니오
Mg	______	○ 정상 ○ 비정상	○ 예 ○ 아니오
Na	______	○ 정상 ○ 비정상	○ 예 ○ 아니오
K	______	○ 정상 ○ 비정상	○ 예 ○ 아니오
Cl	______	○ 정상 ○ 비정상	○ 예 ○ 아니오
P	______	○ 정상 ○ 비정상	○ 예 ○ 아니오
Total cholesterol	______	○ 정상 ○ 비정상	○ 예 ○ 아니오
Triglyceride	______	○ 정상 ○ 비정상	○ 예 ○ 아니오
CPK	______	○ 정상 ○ 비정상	○ 예 ○ 아니오
Glucose	______	○ 정상 ○ 비정상	○ 예 ○ 아니오
CrCl[자동계산]	______		

Urinalysis			
ND	☐ ND		
검사항목	결과	정상 여부	임상적 유의성
Specific gravity	______	○ 정상 ○ 비정상	○ 예 ○ 아니오
pH	______	○ 정상 ○ 비정상	○ 예 ○ 아니오
Bilirubin[U]	______	○ 정상 ○ 비정상	○ 예 ○ 아니오
Urobilinogen[U]	______	○ 정상 ○ 비정상	○ 예 ○ 아니오
Blood[U]	______	○ 정상 ○ 비정상	○ 예 ○ 아니오

검사항목	결과	정상 여부	임상적 유의성
Glucose[U]	______	○ 정상 ○ 비정상	○ 예 ○ 아니오
Protein[U] (Alb[U])	______	○ 정상 ○ 비정상	○ 예 ○ 아니오
Ketones[U]	______	○ 정상 ○ 비정상	○ 예 ○ 아니오
Leukocytes[U]	______	○ 정상 ○ 비정상	○ 예 ○ 아니오

Thyroid Function Tests

ND	☐ ND		
검사항목	결과	정상 여부	임상적 유의성
TSH	______	○ 정상 ○ 비정상	○ 예 ○ 아니오
Free T4	______	○ 정상 ○ 비정상	○ 예 ○ 아니오

Tests for HbA1c

ND	☐ ND		
검사항목	결과	정상 여부	임상적 유의성
HbA1c	______	○ 정상 ○ 비정상	○ 예 ○ 아니오

Coagulation Profile

ND	☐ ND		
검사항목	결과	정상 여부	임상적 유의성
PT INR	______	○ 정상 ○ 비정상	○ 예 ○ 아니오
aPTT	______	○ 정상 ○ 비정상	○ 예 ○ 아니오

12-Lead ECG

ND	☐ ND
미실시인 경우 사유	________<br>________<br>________
검사일	______
측정횟수	○ 1회<br>○ 3회
BBB 여부	○ 예<br>○ 아니오

No.	Heart Rate	PR Interval	QRS Duration	QT Interval	QTcB [자동계산]	QTcF [자동계산]
1차	__ bpm	__ msec	__ msec	__ msec	__.	__.
2차	__ bpm	__ msec	__ msec	__ msec	__.	__.
3차	__ bpm	__ msec	__ msec	__ msec	__.	__.

QTcB 평균[자동계산]	__.
QTcF 평균[자동계산]	__.
임상적으로 유의한 비정상 소견이 있습니까?	○ 예<br>○ 아니오

* 동의서 취득 이후 임상적으로 유의한 비정상 소견이 있을 경우, 비정상 소견은 '이상반응'에 기록하십시오.
* Screen, C1D1 시점에서는 3회 측정하고, 이후 주기에서는 1회만 수행합니다.
(단, ECG를 통해 QT 간격 연장이 입증된 경우, 단기간(즉시)에 추가로 2개의 ECG를 수행하여 최종 3개 ECG의 평균 QTc interval을 확인합니다.)

ECHO 또는 MUGA scan	
ND	☐ ND
미실시인 경우 사유	____________<br>____________<br>____________
검사일	______
검사 방법	○ ECHO<br>○ MUGA scan
좌심실구출률(LVEF)	__._ %
임상적으로 유의한 비정상 소견이 있습니까?	○ 예<br>○ 아니오
* 동의서 취득 이후 임상적으로 유의한 비정상 소견이 있을 경우, 비정상 소견은 ‘이상반응’에 기록하십시오.	

시험대상자 적합성	
상기 시험대상자는 선정/제외 기준을 만족하고, 임상시험 참여에 적합합니까?	○ 예<br>○ 아니오
탈락사유	○ 선정/제외기준 부적합 → Specify below<br>○ 시험대상자 동의철회<br>○ 기타 → Specify below
탈락사유 상세	__________<br>__________<br>__________

선정기준
대상자는 다음의 모든 기준에 적격하여야 본 임상에 참여할 수 있다.
1. 서면동의일 기준으로 만 19세 이상의 여성
2. 임신(임신가능성, 임신계획), 수유 여부<br>① 비가임 여성, 임신 또는 수유 중이 아닌 여성, 임상시험 기간 동안 임신계획이 없는 여성<br>② 가임 여성인 경우, 스크리닝 기간 및 PHI-101 투여 직전의 임신반응검사에서 음성으로 확인되고, 본 계획서에서 요구하는 효과적인 피임법을 PHI-101의 마지막 투여 후 6개월(24주)간 준수하기로 동의한 경우
3. 대상질환<br>① 조직학적(histologically) 또는 세포학적(cytologically)으로 확인된 난소암, 난관암 또는 원발성 복막암<br>② 국소조직학적 소견(local histopathological findings)으로 진단된 상피성암(epithelial malignant tumors) [WHO Histological Classification, 2014]<br>: 단, 흔하지 않는 난소 조직병리(LCOH)로 분류되는, 저등급장액성난소암(LGSC), 점액성난소암(mucinous carcinoma of the ovary), 암육종(악성혼합뮐러종양[MMMT]), 경계성상피성난소종양(저악성암종[LAP]) 제외 [NCCN Guideline version 2.2019]<br>③ 백금불응성암* 또는 백금저항성암**<br>*백금기반 항암요법 진행중, 질병이 진행함, **백금기반 항암요법 완료 후, 6개월(24주) 이내에 질병이 진행함<br>④ 현재 임상적 이익이 있는 것으로 알려진 표준치료법에 대해 불응하거나, 받을 수 없거나, 거부한 자로 수술이 불가한 경우<br>⑤ [RECIST version 1.1]에 부합하는, 최소 한 개 이상의 측정 가능한 병변 또는 측정 불가하나 평가 가능한 병변을 가진 경우
4. 예상 생존기간 ≥ 12주
5. [ECOG PS] ≤ 1

6. 다음의 실험실적 검사를 통해 적절한 간, 신장, 혈액학적 기능이 확인된 자(스크리닝 기간 동안 재검 가능하다)
① ANC ≥ 1,500/mm³ (단, 베이스라인 이전 2주 이내에 G-CSF 투여 없이)
② Hb ≥ 10.0 g/dL (단, 베이스라인 이전 2주 이내에 수혈 없이)
③ Platelet count ≥ 75,000/mm³ (단, 베이스라인 이전 2주 이내에 수혈 없이)
④ Total bilirubin ≤ 1.5 x ULN
⑤ AST and ALT ≤ 3.0 x ULN* (단, 간전이 또는 간세포암인 경우 ≤ 5 x ULN)
⑥ Serum creatinine ≤ 1.5 x ULN (or CrCl ≥ 60 mL/min (by Cockcroft-Gault equation)

7. 이전 항암요법 및 치료
① 이전 항암화학요법 ≤ 5 회
② 이전 항암요법(수술, 약물, 방사선치료 등)*들의 가역적인 부작용들(reversible side effects)로부터 [CTCAE version 5.0] grade 1 또는 그보다 양호한 상태로 회복된 경우
*대수술(major surgery), 항암요법(antineoplastic therapy) 또는 실험적인 치료(experimental therapy), 조혈부위의 직접적인 방사선요법은 베이스라인 이전4주 이내에 적용(투여)받지 않았어야 하며, nitrosoureas 또는 mitomycin-C는 베이스라인 이전 6주 이내에 투여 받지 않았어야 함.

8. 본 임상시험에 대해 충분한 설명을 들은 후 자의로 참여를 결정하고 서면 동의한 자

9. 본 임상시험의 절차 및 제한 사항 등을 이해하고, 임상시험 참여기간 동안 이를 준수할 의사가 있는 자

제외기준

대상자는 다음 조건의 어느 하나라도 해당되는 경우 본 임상시험에 참여할 수 없다.

1. PHI-101의 주성분 또는 부형제에 대해 알려진 또는 의심되는 과민반응 또는 불내성(intolerance)이 있는 경우

2. 시험자가 판단하였을 때 본 임상시험 참여가 부적합 또는 불가능한 환자

3. 다음과 같은 심장기능 부전 또는 심혈관계 질환(단, 이에 국한되지 않는다)
① 베이스라인 이전12주 이내에 발생한 심근허혈의 증거 또는 심근경색
② [NYHA Functional Classification] ≥ II
③ ECHO 또는 MUGA scan을 통해 확인된 LVEF ＜ 50%
④ 임상적으로 유의한, 최적의 적절한 요법에 의해 조절되지 않는 심부정맥
⑤ Corrected QT (QTc)* interval ＞ 450 msec (남/녀 동일), Long QT 증후군(또는 이의 가족력)
*Fridericia 공식에 의해 보정된 QT 간격(QTcF)을 이용한다. 단, 각차단(bundle branch block)이 있을 시 Bazett 공식으로 계산한다(QTcB).

4. 다음과 같은 약물 복용 또는 흡수에 영향을 주는 위장관 질환(단, 이에 국한되지 않는다)
① 연하곤란
② 장관마비, 장폐색
③ 약물 흡수에 임상적으로 유의한 영향을 주는 위장관 수술: 위절제술, 소장루, 광범위한 소장절제술 등(단, 단순 문합술은 제외된다)
④ 위장관계 전체 또는 소장을 침범하는 자가면역 및 염증성 질환: 셀리악병(coeliac disease), 장의 이식편대숙주질환, 베체트신드롬, 위장관을 침범한 경피증(scleroderma), 크론병(Crohn's disease), 궤양성 대장염(ulcerative colitis) 등

5. 다음과 같은 폐질환(단, 이에 국한되지 않는다)
① 새로이 시작된 또는 진행성의 호흡곤란, 기침 및 발열 증상이 있는 경우
② 간질성 폐질환 진단 대기 중인 환자 또는 간질성 폐렴
③ 폐섬유증

6. 림프종(lymphoma)을 포함한 혈액암(hematologic malignancy)
7. 다음과 같은 전이 환자<br>① 중추신경계전이 또는 뇌전이<br>② 골전이
8. 다음과 같은 감염질환(단, 이에 국한되지 않는다)<br>① 임상시험기간 중 안전성 및 유효성 평가에 영향을 줄 수 있는 항생제, 항바이러스제 등의 투여를 요하는 중증의 감염질환<br>② 최적의 적절한 요법에 의해 조절되지 않는 활성(현성) 감염질환으로 시험자가 판단하는 경우
9. 알려진 인체 면역결핍 바이러스(HIV) 양성
10. 활동성B 형 간염* 또는 활동성 C 형 간염**<br>*HBsAg 양성이면서 HBV DNA 가 검출됨. **Anti-HCV 양성이면서 HCV RNA가 (정성적으로)검출됨
11. 서면동의일 기준 12주 이내의 의도하지 않은(unintentional) 체중 감소 ＞ 10%인 경우
12. 서면동의일 기준 1년(52주) 이내의 알코올 또는 다른 약물의 남용 이력
13. 임상시험 참여기간 동안 임상시험용 의약품 외의 다른 항암요법*이 필요한 경우(단, 기관지 폐쇄, 피부 병변 등의 완화를 목적으로 하는 국소 방사선 조사(point radiation)는 허용된다).<br>*수술, 방사선(화학)요법, 항암화학요법, 표적요법제(저분자의약품, 단일클론항체), 면역항암제(생물학적제제), 또는 호르몬요법 등.
14. 다른 임상시험용 의약품이나 임상시험용 의료기기를 서면동의일 기준 2주 이내, 또는 이의 반감기의 5배에 해당하는 기간 이내(이중 더 짧은 기간)에 투여(적용) 받은 경우
15. 임상시험용 의약품의 동태(체내에서의 대사 및 배설 등) 또는 유효성과 안전성 평가에 유의한 영향을 미치는 약물(비처방약, 허브 또는 동종요법 등 포함)을 스크리닝 이전 2주 이내 투여 중인 것으로 시험자가 판단하는 경우

PK 채혈	
ND	☐ ND
미실시인 경우 사유	____________________<br>____________________<br>____________________
PHI-101 투약일	______
PHI-101 투약시간	___

채혈 시점	ND	채혈일	채혈 시간
투여 전(-30분)	☐	______	___
투여 후 0.5 시간(±5분)	☐	______	___
투여 후 1 시간(±5분)	☐	______	___
투여 후 2 시간(±0.25시간)	☐	______	___
투여 후 4 시간(±0.5시간)	☐	______	___
투여 후 6 시간(±0.5시간)	☐	______	___
투여 후 8 시간(±0.5시간)	☐	______	___
투여 후 24 시간(±1시간)	☐	______	___

방문일	
방문일	______

선행/병용약물 확인
지난 방문과 비교하였을 때, 새롭게 투여되었거나 투여 용량이 변경된 병용약물이 있는 경우 선행/병용약물 페이지에 기록하여 주십시오.

이상반응 확인
지난 방문과 비교하였을 때, 새롭게 발생한 이상반응이 있을 경우 이상반응 페이지에 기록하여 주십시오.<br>(만약, EOT방문 전 새로운 항암요법이 수행되는 경우에는 해당 시점까지만 이상반응을 수집합니다.)

실험실 검사	
ND	☐ ND
미실시인 경우 사유	____<br>____<br>____
최소 8시간 금식 여부	○ 예<br>○ 아니오
검체 채취일	______

* 동의서 취득 이후 검사 결과가 'Clinical Significance' 일 경우 비정상 소견은 '이상반응'에 기록하십시오.
* 검사 항목별 단위는 "정상 참고 범위"와 함께 제공되므로 아래 결과에 기록하지 않습니다.

Hematology			
ND	☐ ND		
검사항목	결과	정상 여부	임상적 유의성
WBC	______	○ 정상 ○ 비정상	○ 예 ○ 아니오
RBC	______	○ 정상 ○ 비정상	○ 예 ○ 아니오
Hemoglobin	______	○ 정상 ○ 비정상	○ 예 ○ 아니오
Hematocrit	______	○ 정상 ○ 비정상	○ 예 ○ 아니오
Platelet	______	○ 정상 ○ 비정상	○ 예 ○ 아니오
MCV	______	○ 정상 ○ 비정상	○ 예 ○ 아니오
MCH	______	○ 정상 ○ 비정상	○ 예 ○ 아니오
MCHC	______	○ 정상 ○ 비정상	○ 예 ○ 아니오
Neutrophils	______	○ 정상 ○ 비정상	○ 예 ○ 아니오

검사항목	결과	정상 여부	임상적 유의성
Lymphocytes	______	○ 정상 ○ 비정상	○ 예 ○ 아니오
Monocytes	______	○ 정상 ○ 비정상	○ 예 ○ 아니오
Eosinophils	______	○ 정상 ○ 비정상	○ 예 ○ 아니오
Basophils	______	○ 정상 ○ 비정상	○ 예 ○ 아니오
ANC	______	○ 정상 ○ 비정상	○ 예 ○ 아니오

Chemistry			
ND	☐ ND		
검사항목	결과	정상 여부	임상적 유의성
BUN	______	○ 정상 ○ 비정상	○ 예 ○ 아니오
Creatinine	______	○ 정상 ○ 비정상	○ 예 ○ 아니오
Total bilirubin	______	○ 정상 ○ 비정상	○ 예 ○ 아니오
AST	______	○ 정상 ○ 비정상	○ 예 ○ 아니오
ALT	______	○ 정상 ○ 비정상	○ 예 ○ 아니오
ALP	______	○ 정상 ○ 비정상	○ 예 ○ 아니오
ɣGT	______	○ 정상 ○ 비정상	○ 예 ○ 아니오
LDH	______	○ 정상 ○ 비정상	○ 예 ○ 아니오
Total protein	______	○ 정상 ○ 비정상	○ 예 ○ 아니오
Albumin	______	○ 정상 ○ 비정상	○ 예 ○ 아니오
Globulin[자동계산]	______	○ 정상 ○ 비정상	○ 예 ○ 아니오

검사항목	결과	정상 여부	임상적 유의성
Ca	______	○ 정상 ○ 비정상	○ 예 ○ 아니오
Mg	______	○ 정상 ○ 비정상	○ 예 ○ 아니오
Na	______	○ 정상 ○ 비정상	○ 예 ○ 아니오
K	______	○ 정상 ○ 비정상	○ 예 ○ 아니오
Cl	______	○ 정상 ○ 비정상	○ 예 ○ 아니오
P	______	○ 정상 ○ 비정상	○ 예 ○ 아니오
Total cholesterol	______	○ 정상 ○ 비정상	○ 예 ○ 아니오
Triglyceride	______	○ 정상 ○ 비정상	○ 예 ○ 아니오
CPK	______	○ 정상 ○ 비정상	○ 예 ○ 아니오
Glucose	______	○ 정상 ○ 비정상	○ 예 ○ 아니오
CrCl[자동계산]	______		

ECHO 또는 MUGA scan	
ND	☐ ND
미실시인 경우 사유	______<br>______<br>______
검사일	______
검사 방법	○ ECHO<br>○ MUGA scan
좌심실구출률(LVEF)	__._ %
임상적으로 유의한 비정상 소견이 있습니까?	○ 예<br>○ 아니오
* 동의서 취득 이후 임상적으로 유의한 비정상 소견이 있을 경우, 비정상 소견은 ‘이상반응’에 기록하십시오.	

PK 채혈	
ND	☐ ND
미실시인 경우 사유	___<br>___<br>___
PHI-101 투약일	___
PHI-101 투약시간	___

채혈 시점	ND	채혈일	채혈 시간
투여 전(-30분)	☐	___	___

방문일	
방문일	______

선행/병용약물 확인
지난 방문과 비교하였을 때, 새롭게 투여되었거나 투여 용량이 변경된 병용약물이 있는 경우 선행/병용약물 페이지에 기록하여 주십시오.

이상반응 확인
지난 방문과 비교하였을 때, 새롭게 발생한 이상반응이 있을 경우 이상반응 페이지에 기록하여 주십시오.<br>(만약, EOT방문 전 새로운 항암요법이 수행되는 경우에는 해당 시점까지만 이상반응을 수집합니다.)

활력징후	
ND	☐ ND
미실시인 경우 사유	________________<br>________________<br>________________
5분 이상 안정을 취한 후 앉은 자세(좌위)에서 측정합니다.	
측정일	______
수축기 혈압	__ mmHg
이완기 혈압	__ mmHg
맥박	__ 회/분
호흡수	__ 회/분
체온	__._ ℃
임상적으로 유의한 비정상 소견이 있습니까?	○ 예<br>○ 아니오
동의서 취득 이후 임상적으로 유의한 비정상 소견이 있을 경우, 비정상 소견은 '이상반응'에 기록하십시오.	

신체검진	
ND	☐ ND
미실시인 경우 사유	______
검진일	______
지난 방문과 비교하였을 때, 각 신체기관 검진 결과 새로운 비정상 소견이 있습니까?	○ 예 → 아래 표에 신체기관 및 상세내용을 기재하여 주십시오.<br>○ 아니오

동의서 취득 이후 임상적으로 유의한 비정상 소견이 있을 경우, 비정상 소견은 '이상반응'에 기록하십시오.

No	신체기관	임상적 유의성
1	___▼	○ 예 ○ 아니오

신체기관	외관 , 피부 , 두/경부 , 흉부/폐 , 심장 , 복부 , 비뇨/생식계 , 사지 , 근골격계 , 신경계 , 림프절 , 기타
신체기관_기타	______

ECOG PS	
ND	☐ ND
미실시인 경우 사유	____________________<br>____________________<br>____________________
평가일	______
ECOG PS	○ Grade 0<br>○ Grade 1<br>○ Grade 2<br>○ Grade 3<br>○ Grade 4<br>○ Grade 5

실험실 검사

ND	☐ ND
미실시인 경우 사유	______<br>______<br>______
최소 8시간 금식 여부	○ 예<br>○ 아니오
검체 채취일	______

* 동의서 취득 이후 검사 결과가 ‘Clinical Significance’ 일 경우 비정상 소견은 ‘이상반응’에 기록하십시오.
* 검사 항목별 단위는 “정상 참고 범위”와 함께 제공되므로 아래 결과에 기록하지 않습니다.

Hematology

ND	☐ ND

검사항목	결과	정상 여부	임상적 유의성
WBC	______	○ 정상 ○ 비정상	○ 예 ○ 아니오
RBC	______	○ 정상 ○ 비정상	○ 예 ○ 아니오
Hemoglobin	______	○ 정상 ○ 비정상	○ 예 ○ 아니오
Hematocrit	______	○ 정상 ○ 비정상	○ 예 ○ 아니오
Platelet	______	○ 정상 ○ 비정상	○ 예 ○ 아니오
MCV	______	○ 정상 ○ 비정상	○ 예 ○ 아니오
MCH	______	○ 정상 ○ 비정상	○ 예 ○ 아니오
MCHC	______	○ 정상 ○ 비정상	○ 예 ○ 아니오
Neutrophils	______	○ 정상 ○ 비정상	○ 예 ○ 아니오

검사항목	결과	정상 여부	임상적 유의성
Lymphocytes	______	○ 정상 ○ 비정상	○ 예 ○ 아니오
Monocytes	______	○ 정상 ○ 비정상	○ 예 ○ 아니오
Eosinophils	______	○ 정상 ○ 비정상	○ 예 ○ 아니오
Basophils	______	○ 정상 ○ 비정상	○ 예 ○ 아니오
ANC	______	○ 정상 ○ 비정상	○ 예 ○ 아니오

Chemistry			
ND	☐ ND		
검사항목	결과	정상 여부	임상적 유의성
BUN	______	○ 정상 ○ 비정상	○ 예 ○ 아니오
Creatinine	______	○ 정상 ○ 비정상	○ 예 ○ 아니오
Total bilirubin	______	○ 정상 ○ 비정상	○ 예 ○ 아니오
AST	______	○ 정상 ○ 비정상	○ 예 ○ 아니오
ALT	______	○ 정상 ○ 비정상	○ 예 ○ 아니오
ALP	______	○ 정상 ○ 비정상	○ 예 ○ 아니오
ɣGT	______	○ 정상 ○ 비정상	○ 예 ○ 아니오
LDH	______	○ 정상 ○ 비정상	○ 예 ○ 아니오
Total protein	______	○ 정상 ○ 비정상	○ 예 ○ 아니오
Albumin	______	○ 정상 ○ 비정상	○ 예 ○ 아니오
Globulin[자동계산]	______	○ 정상 ○ 비정상	○ 예 ○ 아니오

검사항목	결과	정상 여부	임상적 유의성
Ca	______	○ 정상 ○ 비정상	○ 예 ○ 아니오
Mg	______	○ 정상 ○ 비정상	○ 예 ○ 아니오
Na	______	○ 정상 ○ 비정상	○ 예 ○ 아니오
K	______	○ 정상 ○ 비정상	○ 예 ○ 아니오
Cl	______	○ 정상 ○ 비정상	○ 예 ○ 아니오
P	______	○ 정상 ○ 비정상	○ 예 ○ 아니오
Total cholesterol	______	○ 정상 ○ 비정상	○ 예 ○ 아니오
Triglyceride	______	○ 정상 ○ 비정상	○ 예 ○ 아니오
CPK	______	○ 정상 ○ 비정상	○ 예 ○ 아니오
Glucose	______	○ 정상 ○ 비정상	○ 예 ○ 아니오
CrCl[자동계산]	______		

ECHO 또는 MUGA scan	
ND	☐ ND
미실시인 경우 사유	____________<br>____________<br>____________
검사일	______
검사 방법	○ ECHO<br>○ MUGA scan
좌심실구출률(LVEF)	__. %
임상적으로 유의한 비정상 소견이 있습니까?	○ 예<br>○ 아니오
* 동의서 취득 이후 임상적으로 유의한 비정상 소견이 있을 경우, 비정상 소견은 '이상반응'에 기록하십시오.	

PK 채혈	
ND	☐ ND
미실시인 경우 사유	____________<br>____________<br>____________
PHI-101 투약일	______
PHI-101 투약시간	___

채혈 시점	ND	채혈일	채혈 시간
투여 전(-30분)	☐	______	___
투여 후 0.5 시간(±5분)	☐	______	___
투여 후 1 시간(±5분)	☐	______	___
투여 후 2 시간(±0.25시간)	☐	______	___
투여 후 4 시간(±0.5시간)	☐	______	___
투여 후 6 시간(±0.5시간)	☐	______	___
투여 후 8 시간(±0.5시간)	☐	______	___
투여 후 24 시간(±1시간)	☐	______	___

방문일

방문일	______

선행/병용약물 확인

지난 방문과 비교하였을 때, 새롭게 투여되었거나 투여 용량이 변경된 병용약물이 있는 경우 선행/병용약물 페이지에 기록하여 주십시오.

이상반응 확인

지난 방문과 비교하였을 때, 새롭게 발생한 이상반응이 있을 경우 이상반응 페이지에 기록하여 주십시오.
(만약, EOT방문 전 새로운 항암요법이 수행되는 경우에는 해당 시점까지만 이상반응을 수집합니다.)

실험실 검사

ND	☐ ND
미실시인 경우 사유	______<br>______<br>______
최소 8시간 금식 여부	○ 예<br>○ 아니오
검체 채취일	______

* 동의서 취득 이후 검사 결과가 'Clinical Significance' 일 경우 비정상 소견은 '이상반응'에 기록하십시오.
* 검사 항목별 단위는 "정상 참고 범위"와 함께 제공되므로 아래 결과에 기록하지 않습니다.

Hematology

ND	☐ ND

검사항목	결과	정상 여부	임상적 유의성
WBC	______	○ 정상 ○ 비정상	○ 예 ○ 아니오
RBC	______	○ 정상 ○ 비정상	○ 예 ○ 아니오
Hemoglobin	______	○ 정상 ○ 비정상	○ 예 ○ 아니오
Hematocrit	______	○ 정상 ○ 비정상	○ 예 ○ 아니오
Platelet	______	○ 정상 ○ 비정상	○ 예 ○ 아니오
MCV	______	○ 정상 ○ 비정상	○ 예 ○ 아니오
MCH	______	○ 정상 ○ 비정상	○ 예 ○ 아니오
MCHC	______	○ 정상 ○ 비정상	○ 예 ○ 아니오
Neutrophils	______	○ 정상 ○ 비정상	○ 예 ○ 아니오

검사항목	결과	정상 여부	임상적 유의성
Lymphocytes	______	○ 정상 ○ 비정상	○ 예 ○ 아니오
Monocytes	______	○ 정상 ○ 비정상	○ 예 ○ 아니오
Eosinophils	______	○ 정상 ○ 비정상	○ 예 ○ 아니오
Basophils	______	○ 정상 ○ 비정상	○ 예 ○ 아니오
ANC	______	○ 정상 ○ 비정상	○ 예 ○ 아니오

Chemistry			
ND	☐ ND		
검사항목	결과	정상 여부	임상적 유의성
BUN	______	○ 정상 ○ 비정상	○ 예 ○ 아니오
Creatinine	______	○ 정상 ○ 비정상	○ 예 ○ 아니오
Total bilirubin	______	○ 정상 ○ 비정상	○ 예 ○ 아니오
AST	______	○ 정상 ○ 비정상	○ 예 ○ 아니오
ALT	______	○ 정상 ○ 비정상	○ 예 ○ 아니오
ALP	______	○ 정상 ○ 비정상	○ 예 ○ 아니오
ɣGT	______	○ 정상 ○ 비정상	○ 예 ○ 아니오
LDH	______	○ 정상 ○ 비정상	○ 예 ○ 아니오
Total protein	______	○ 정상 ○ 비정상	○ 예 ○ 아니오
Albumin	______	○ 정상 ○ 비정상	○ 예 ○ 아니오
Globulin[자동계산]	______	○ 정상 ○ 비정상	○ 예 ○ 아니오

검사항목	결과	정상 여부	임상적 유의성
Ca	______	○ 정상 ○ 비정상	○ 예 ○ 아니오
Mg	______	○ 정상 ○ 비정상	○ 예 ○ 아니오
Na	______	○ 정상 ○ 비정상	○ 예 ○ 아니오
K	______	○ 정상 ○ 비정상	○ 예 ○ 아니오
Cl	______	○ 정상 ○ 비정상	○ 예 ○ 아니오
P	______	○ 정상 ○ 비정상	○ 예 ○ 아니오
Total cholesterol	______	○ 정상 ○ 비정상	○ 예 ○ 아니오
Triglyceride	______	○ 정상 ○ 비정상	○ 예 ○ 아니오
CPK	______	○ 정상 ○ 비정상	○ 예 ○ 아니오
Glucose	______	○ 정상 ○ 비정상	○ 예 ○ 아니오
CrCl[자동계산]	______		

ECHO 또는 MUGA scan	
ND	☐ ND
미실시인 경우 사유	__________<br>__________<br>__________
검사일	______
검사 방법	○ ECHO<br>○ MUGA scan
좌심실구출률(LVEF)	__._ %
임상적으로 유의한 비정상 소견이 있습니까?	○ 예<br>○ 아니오
* 동의서 취득 이후 임상적으로 유의한 비정상 소견이 있을 경우, 비정상 소견은 '이상반응'에 기록하십시오.	

PK 채혈	
ND	☐ ND
미실시인 경우 사유	____________<br>____________<br>____________
PHI-101 투약일	______
PHI-101 투약시간	___

채혈 시점	ND	채혈일	채혈 시간
투여 전(-30분)	☐	______	___

방문일	
방문일	______

선행/병용약물 확인
지난 방문과 비교하였을 때, 새롭게 투여되었거나 투여 용량이 변경된 병용약물이 있는 경우 선행/병용약물 페이지에 기록하여 주십시오.

이상반응 확인
지난 방문과 비교하였을 때, 새롭게 발생한 이상반응이 있을 경우 이상반응 페이지에 기록하여 주십시오.<br>(만약, EOT방문 전 새로운 항암요법이 수행되는 경우에는 해당 시점까지만 이상반응을 수집합니다.)

**활력징후**	
ND	☐ ND
미실시인 경우 사유	______________________<br>______________________<br>______________________
5분 이상 안정을 취한 후 앉은 자세(좌위)에서 측정합니다.	
측정일	______
수축기 혈압	__ mmHg
이완기 혈압	__ mmHg
맥박	__ 회/분
호흡수	__ 회/분
체온	__. ℃
임상적으로 유의한 비정상 소견이 있습니까?	○ 예<br>○ 아니오
동의서 취득 이후 임상적으로 유의한 비정상 소견이 있을 경우, 비정상 소견은 '이상반응'에 기록하십시오.	

**신체계측**	
ND	☐ ND
미실시인 경우 사유	______________________<br>______________________<br>______________________
측정일	______
체중	___. kg

신체검진	
ND	☐ ND
미실시인 경우 사유	______________________________<br>______________________________<br>______________________________
검진일	______
지난 방문과 비교하였을 때, 각 신체기관 검진 결과 새로운 비정상 소견이 있습니까?	○ 예 → 아래 표에 신체기관 및 상세내용을 기재하여 주십시오.<br>○ 아니오

동의서 취득 이후 임상적으로 유의한 비정상 소견이 있을 경우, 비정상 소견은 '이상반응'에 기록하십시오.

No	신체기관	임상적 유의성
1	___▼	○ 예 ○ 아니오

신체기관	외관 , 피부 , 두/경부 , 흉부/폐 , 심장 , 복부 , 비뇨/생식계 , 사지 , 근골격계 , 신경계 , 림프절 , 기타
신체기관_기타	______________________________<br>______________________________<br>______________________________

ECOG PS	
ND	☐ ND
미실시인 경우 사유	______________________________<br>______________________________<br>______________________________
평가일	______
ECOG PS	○ Grade 0<br>○ Grade 1<br>○ Grade 2<br>○ Grade 3<br>○ Grade 4<br>○ Grade 5

임신검사	
ND	☐ ND
미실시인 경우 사유	________________<br>________________<br>________________
검사일	______
검사 방법	○ urine-hCG<br>○ serum-hCG
검사결과	○ 양성<br>○ 음성
urine-hCG(및/또는 serum hCG) 검사를 실시합니다.	

실험실 검사	
ND	☐ ND
미실시인 경우 사유	______<br>______<br>______
최소 8시간 금식 여부	○ 예<br>○ 아니오
검체 채취일	______

* 동의서 취득 이후 검사 결과가 'Clinical Significance' 일 경우 비정상 소견은 '이상반응'에 기록하십시오.
* 검사 항목별 단위는 "정상 참고 범위"와 함께 제공되므로 아래 결과에 기록하지 않습니다.

Hematology			
ND	☐ ND		
검사항목	결과	정상 여부	임상적 유의성
WBC	______	○ 정상 ○ 비정상	○ 예 ○ 아니오
RBC	______	○ 정상 ○ 비정상	○ 예 ○ 아니오
Hemoglobin	______	○ 정상 ○ 비정상	○ 예 ○ 아니오
Hematocrit	______	○ 정상 ○ 비정상	○ 예 ○ 아니오
Platelet	______	○ 정상 ○ 비정상	○ 예 ○ 아니오
MCV	______	○ 정상 ○ 비정상	○ 예 ○ 아니오
MCH	______	○ 정상 ○ 비정상	○ 예 ○ 아니오
MCHC	______	○ 정상 ○ 비정상	○ 예 ○ 아니오
Neutrophils	______	○ 정상 ○ 비정상	○ 예 ○ 아니오

검사항목	결과	정상 여부	임상적 유의성
Lymphocytes	______	○ 정상 ○ 비정상	○ 예 ○ 아니오
Monocytes	______	○ 정상 ○ 비정상	○ 예 ○ 아니오
Eosinophils	______	○ 정상 ○ 비정상	○ 예 ○ 아니오
Basophils	______	○ 정상 ○ 비정상	○ 예 ○ 아니오
ANC	______	○ 정상 ○ 비정상	○ 예 ○ 아니오

Chemistry			
ND	☐ ND		
검사항목	결과	정상 여부	임상적 유의성
BUN	______	○ 정상 ○ 비정상	○ 예 ○ 아니오
Creatinine	______	○ 정상 ○ 비정상	○ 예 ○ 아니오
Total bilirubin	______	○ 정상 ○ 비정상	○ 예 ○ 아니오
AST	______	○ 정상 ○ 비정상	○ 예 ○ 아니오
ALT	______	○ 정상 ○ 비정상	○ 예 ○ 아니오
ALP	______	○ 정상 ○ 비정상	○ 예 ○ 아니오
ɣGT	______	○ 정상 ○ 비정상	○ 예 ○ 아니오
LDH	______	○ 정상 ○ 비정상	○ 예 ○ 아니오
Total protein	______	○ 정상 ○ 비정상	○ 예 ○ 아니오
Albumin	______	○ 정상 ○ 비정상	○ 예 ○ 아니오
Globulin[자동계산]	______	○ 정상 ○ 비정상	○ 예 ○ 아니오

검사항목	결과	정상 여부	임상적 유의성
Ca	______	○ 정상 ○ 비정상	○ 예 ○ 아니오
Mg	______	○ 정상 ○ 비정상	○ 예 ○ 아니오
Na	______	○ 정상 ○ 비정상	○ 예 ○ 아니오
K	______	○ 정상 ○ 비정상	○ 예 ○ 아니오
Cl	______	○ 정상 ○ 비정상	○ 예 ○ 아니오
P	______	○ 정상 ○ 비정상	○ 예 ○ 아니오
Total cholesterol	______	○ 정상 ○ 비정상	○ 예 ○ 아니오
Triglyceride	______	○ 정상 ○ 비정상	○ 예 ○ 아니오
CPK	______	○ 정상 ○ 비정상	○ 예 ○ 아니오
Glucose	______	○ 정상 ○ 비정상	○ 예 ○ 아니오
CrCl[자동계산]	______		

Urinalysis			
ND	☐ ND		
검사항목	결과	정상 여부	임상적 유의성
Specific gravity	______	○ 정상 ○ 비정상	○ 예 ○ 아니오
pH	______	○ 정상 ○ 비정상	○ 예 ○ 아니오
Bilirubin[U]	______	○ 정상 ○ 비정상	○ 예 ○ 아니오
Urobilinogen[U]	______	○ 정상 ○ 비정상	○ 예 ○ 아니오
Blood[U]	______	○ 정상 ○ 비정상	○ 예 ○ 아니오

검사항목	결과	정상 여부	임상적 유의성
Glucose[U]	______	○ 정상 ○ 비정상	○ 예 ○ 아니오
Protein[U] (Alb[U])	______	○ 정상 ○ 비정상	○ 예 ○ 아니오
Ketones[U]	______	○ 정상 ○ 비정상	○ 예 ○ 아니오
Leukocytes[U]	______	○ 정상 ○ 비정상	○ 예 ○ 아니오

12-Lead ECG

ND	☐ ND
미실시인 경우 사유	______<br>______<br>______
검사일	______
측정횟수	○ 1회<br>○ 3회
BBB 여부	○ 예<br>○ 아니오

No.	Heart Rate	PR Interval	QRS Duration	QT Interval	QTcB [자동계산]	QTcF [자동계산]
1차	__ bpm	__ msec	__ msec	__ msec	__.__	__.__
2차	__ bpm	__ msec	__ msec	__ msec	__.__	__.__
3차	__ bpm	__ msec	__ msec	__ msec	__.__	__.__

QTcB 평균[자동계산]	__.__
QTcF 평균[자동계산]	__.__
임상적으로 유의한 비정상 소견이 있습니까?	○ 예<br>○ 아니오

* 동의서 취득 이후 임상적으로 유의한 비정상 소견이 있을 경우, 비정상 소견은 '이상반응'에 기록하십시오.
* Screen, C1D1 시점에서는 3회 측정하고, 이후 주기에서는 1회만 수행합니다.
(단, ECG를 통해 QT 간격 연장이 입증된 경우, 단기간(즉시)에 추가로 2개의 ECG를 수행하여 최종 3개 ECG의 평균 QTc interval을 확인합니다.)

ECHO 또는 MUGA scan	
ND	☐ ND
미실시인 경우 사유	________<br>________<br>________
검사일	______
검사 방법	○ ECHO<br>○ MUGA scan
좌심실구출률(LVEF)	__._ %
임상적으로 유의한 비정상 소견이 있습니까?	○ 예<br>○ 아니오
* 동의서 취득 이후 임상적으로 유의한 비정상 소견이 있을 경우, 비정상 소견은 '이상반응'에 기록하십시오.	

PK 채혈

ND	☐ ND
미실시인 경우 사유	___<br>___<br>___
PHI-101 투약일	______
PHI-101 투약시간	___

채혈 시점	ND	채혈일	채혈 시간
투여 전(-30분)	☐	______	___
투여 후 2~4시간	☐	______	___

방문일

방문일	______

선행/병용약물 확인

지난 방문과 비교하였을 때, 새롭게 투여되었거나 투여 용량이 변경된 병용약물이 있는 경우 선행/병용약물 페이지에 기록하여 주십시오.

이상반응 확인

지난 방문과 비교하였을 때, 새롭게 발생한 이상반응이 있을 경우 이상반응 페이지에 기록하여 주십시오.
(만약, EOT방문 전 새로운 항암요법이 수행되는 경우에는 해당 시점까지만 이상반응을 수집합니다.)

활력징후	
ND	☐ ND
미실시인 경우 사유	____________<br>____________<br>____________
5분 이상 안정을 취한 후 앉은 자세(좌위)에서 측정합니다.	
측정일	______
수축기 혈압	__ mmHg
이완기 혈압	__ mmHg
맥박	__ 회/분
호흡수	__ 회/분
체온	__.__ ℃
임상적으로 유의한 비정상 소견이 있습니까?	○ 예<br>○ 아니오
동의서 취득 이후 임상적으로 유의한 비정상 소견이 있을 경우, 비정상 소견은 '이상반응'에 기록하십시오.	

신체검진

ND	☐ ND
미실시인 경우 사유	________________<br>________________<br>________________
검진일	______
지난 방문과 비교하였을 때, 각 신체기관 검진 결과 새로운 비정상 소견이 있습니까?	○ 예 → 아래 표에 신체기관 및 상세내용을 기재하여 주십시오.<br>○ 아니오

동의서 취득 이후 임상적으로 유의한 비정상 소견이 있을 경우, 비정상 소견은 '이상반응'에 기록하십시오.

No	신체기관	임상적 유의성
1	___▼	○ 예 ○ 아니오

신체기관	외관 , 피부 , 두/경부 , 흉부/폐 , 심장 , 복부 , 비뇨/생식계 , 사지 , 근골격계 , 신경계 , 림프절 , 기타
신체기관_기타	________________<br>________________<br>________________

ECOG PS	
ND	☐ ND
미실시인 경우 사유	____________________<br>____________________<br>____________________
평가일	______
ECOG PS	○ Grade 0<br>○ Grade 1<br>○ Grade 2<br>○ Grade 3<br>○ Grade 4<br>○ Grade 5

실험실 검사	
ND	☐ ND
미실시인 경우 사유	______ ______ ______
최소 8시간 금식 여부	○ 예<br>○ 아니오
검체 채취일	______

* 동의서 취득 이후 검사 결과가 'Clinical Significance' 일 경우 비정상 소견은 '이상반응'에 기록하십시오.
* 검사 항목별 단위는 "정상 참고 범위"와 함께 제공되므로 아래 결과에 기록하지 않습니다.

Hematology			
ND	☐ ND		
검사항목	결과	정상 여부	임상적 유의성
WBC	______	○ 정상 ○ 비정상	○ 예 ○ 아니오
RBC	______	○ 정상 ○ 비정상	○ 예 ○ 아니오
Hemoglobin	______	○ 정상 ○ 비정상	○ 예 ○ 아니오
Hematocrit	______	○ 정상 ○ 비정상	○ 예 ○ 아니오
Platelet	______	○ 정상 ○ 비정상	○ 예 ○ 아니오
MCV	______	○ 정상 ○ 비정상	○ 예 ○ 아니오
MCH	______	○ 정상 ○ 비정상	○ 예 ○ 아니오
MCHC	______	○ 정상 ○ 비정상	○ 예 ○ 아니오
Neutrophils	______	○ 정상 ○ 비정상	○ 예 ○ 아니오

검사항목	결과	정상 여부	임상적 유의성
Lymphocytes	______	○ 정상 ○ 비정상	○ 예 ○ 아니오
Monocytes	______	○ 정상 ○ 비정상	○ 예 ○ 아니오
Eosinophils	______	○ 정상 ○ 비정상	○ 예 ○ 아니오
Basophils	______	○ 정상 ○ 비정상	○ 예 ○ 아니오
ANC	______	○ 정상 ○ 비정상	○ 예 ○ 아니오

Chemistry

ND	☐ ND		
검사항목	결과	정상 여부	임상적 유의성
BUN	______	○ 정상 ○ 비정상	○ 예 ○ 아니오
Creatinine	______	○ 정상 ○ 비정상	○ 예 ○ 아니오
Total bilirubin	______	○ 정상 ○ 비정상	○ 예 ○ 아니오
AST	______	○ 정상 ○ 비정상	○ 예 ○ 아니오
ALT	______	○ 정상 ○ 비정상	○ 예 ○ 아니오
ALP	______	○ 정상 ○ 비정상	○ 예 ○ 아니오
ɣGT	______	○ 정상 ○ 비정상	○ 예 ○ 아니오
LDH	______	○ 정상 ○ 비정상	○ 예 ○ 아니오
Total protein	______	○ 정상 ○ 비정상	○ 예 ○ 아니오
Albumin	______	○ 정상 ○ 비정상	○ 예 ○ 아니오
Globulin[자동계산]	______	○ 정상 ○ 비정상	○ 예 ○ 아니오

검사항목	결과	정상 여부	임상적 유의성
Ca	______	○ 정상 ○ 비정상	○ 예 ○ 아니오
Mg	______	○ 정상 ○ 비정상	○ 예 ○ 아니오
Na	______	○ 정상 ○ 비정상	○ 예 ○ 아니오
K	______	○ 정상 ○ 비정상	○ 예 ○ 아니오
Cl	______	○ 정상 ○ 비정상	○ 예 ○ 아니오
P	______	○ 정상 ○ 비정상	○ 예 ○ 아니오
Total cholesterol	______	○ 정상 ○ 비정상	○ 예 ○ 아니오
Triglyceride	______	○ 정상 ○ 비정상	○ 예 ○ 아니오
CPK	______	○ 정상 ○ 비정상	○ 예 ○ 아니오
Glucose	______	○ 정상 ○ 비정상	○ 예 ○ 아니오
CrCl[자동계산]	______		

12-Lead ECG

ND	☐ ND
미실시인 경우 사유	______<br>______<br>______
검사일	______
측정횟수	○ 1회<br>○ 3회
BBB 여부	○ 예<br>○ 아니오

No.	Heart Rate	PR Interval	QRS Duration	QT Interval	QTcB [자동계산]	QTcF [자동계산]
1차	__ bpm	__ msec	__ msec	__ msec	__.__	__.__
2차	__ bpm	__ msec	__ msec	__ msec	__.__	__.__
3차	__ bpm	__ msec	__ msec	__ msec	__.__	__.__

QTcB 평균[자동계산]	__.__
QTcF 평균[자동계산]	__.__
임상적으로 유의한 비정상 소견이 있습니까?	○ 예<br>○ 아니오

* 동의서 취득 이후 임상적으로 유의한 비정상 소견이 있을 경우, 비정상 소견은 '이상반응'에 기록하십시오.
* Screen, C1D1 시점에서는 3회 측정하고, 이후 주기에서는 1회만 수행합니다.
(단, ECG를 통해 QT 간격 연장이 입증된 경우, 단기간(즉시)에 추가로 2개의 ECG를 수행하여 최종 3개 ECG의 평균 QTc interval을 확인합니다.)

ECHO 또는 MUGA scan	
ND	☐ ND
미실시인 경우 사유	____________________<br>____________________<br>____________________
검사일	______
검사 방법	○ ECHO<br>○ MUGA scan
좌심실구출률(LVEF)	__._ %
임상적으로 유의한 비정상 소견이 있습니까?	○ 예<br>○ 아니오
* 동의서 취득 이후 임상적으로 유의한 비정상 소견이 있을 경우, 비정상 소견은 ‘이상반응’에 기록하십시오.	

방문일	
방문일	______

선행/병용약물 확인
지난 방문과 비교하였을 때, 새롭게 투여되었거나 투여 용량이 변경된 병용약물이 있는 경우 선행/병용약물 페이지에 기록하여 주십시오.

이상반응 확인
지난 방문과 비교하였을 때, 새롭게 발생한 이상반응이 있을 경우 이상반응 페이지에 기록하여 주십시오.<br>(만약, EOT방문 전 새로운 항암요법이 수행되는 경우에는 해당 시점까지만 이상반응을 수집합니다.)

활력징후	
ND	☐ ND
미실시인 경우 사유	______________________________<br>______________________________<br>______________________________
5분 이상 안정을 취한 후 앉은 자세(좌위)에서 측정합니다.	
측정일	______
수축기 혈압	__ mmHg
이완기 혈압	__ mmHg
맥박	__ 회/분
호흡수	__ 회/분
체온	__._ ℃
임상적으로 유의한 비정상 소견이 있습니까?	○ 예<br>○ 아니오
동의서 취득 이후 임상적으로 유의한 비정상 소견이 있을 경우, 비정상 소견은 '이상반응'에 기록하십시오.	

신체계측	
ND	☐ ND
미실시인 경우 사유	______________________________<br>______________________________<br>______________________________
측정일	______
체중	___._ kg

신체검진

ND	☐ ND
미실시인 경우 사유	______<br>______<br>______
검진일	______
지난 방문과 비교하였을 때, 각 신체기관 검진 결과 새로운 비정상 소견이 있습니까?	○ 예 → 아래 표에 신체기관 및 상세내용을 기재하여 주십시오.<br>○ 아니오

동의서 취득 이후 임상적으로 유의한 비정상 소견이 있을 경우, 비정상 소견은 '이상반응'에 기록하십시오.

No	신체기관	임상적 유의성
1	___▼	○ 예 ○ 아니오

신체기관	외관 , 피부 , 두/경부 , 흉부/폐 , 심장 , 복부 , 비뇨/생식계 , 사지 , 근골격계 , 신경계 , 림프절 , 기타
신체기관_기타	______<br>______<br>______

ECOG PS	
ND	☐ ND
미실시인 경우 사유	______<br>______<br>______
평가일	______
ECOG PS	○ Grade 0<br>○ Grade 1<br>○ Grade 2<br>○ Grade 3<br>○ Grade 4<br>○ Grade 5

임신검사	
ND	☐ ND
미실시인 경우 사유	______________________________<br>______________________________<br>______________________________
검사일	______
검사 방법	○ urine-hCG<br>○ serum-hCG
검사결과	○ 양성<br>○ 음성
urine-hCG(및/또는 serum hCG) 검사를 실시합니다.	

실험실 검사	
ND	☐ ND
미실시인 경우 사유	________________ ________________ ________________
최소 8시간 금식 여부	○ 예 ○ 아니오
검체 채취일	______

* 동의서 취득 이후 검사 결과가 ‘Clinical Significance’ 일 경우 비정상 소견은 ‘이상반응’에 기록하십시오.
* 검사 항목별 단위는 “정상 참고 범위”와 함께 제공되므로 아래 결과에 기록하지 않습니다.

Hematology

ND: ☐ ND

검사항목	결과	정상 여부	임상적 유의성
WBC	______	○ 정상 ○ 비정상	○ 예 ○ 아니오
RBC	______	○ 정상 ○ 비정상	○ 예 ○ 아니오
Hemoglobin	______	○ 정상 ○ 비정상	○ 예 ○ 아니오
Hematocrit	______	○ 정상 ○ 비정상	○ 예 ○ 아니오
Platelet	______	○ 정상 ○ 비정상	○ 예 ○ 아니오
MCV	______	○ 정상 ○ 비정상	○ 예 ○ 아니오
MCH	______	○ 정상 ○ 비정상	○ 예 ○ 아니오
MCHC	______	○ 정상 ○ 비정상	○ 예 ○ 아니오
Neutrophils	______	○ 정상 ○ 비정상	○ 예 ○ 아니오

검사항목	결과	정상 여부	임상적 유의성
Lymphocytes	______	○ 정상 ○ 비정상	○ 예 ○ 아니오
Monocytes	______	○ 정상 ○ 비정상	○ 예 ○ 아니오
Eosinophils	______	○ 정상 ○ 비정상	○ 예 ○ 아니오
Basophils	______	○ 정상 ○ 비정상	○ 예 ○ 아니오
ANC	______	○ 정상 ○ 비정상	○ 예 ○ 아니오

Chemistry			
ND	☐ ND		
검사항목	결과	정상 여부	임상적 유의성
BUN	______	○ 정상 ○ 비정상	○ 예 ○ 아니오
Creatinine	______	○ 정상 ○ 비정상	○ 예 ○ 아니오
Total bilirubin	______	○ 정상 ○ 비정상	○ 예 ○ 아니오
AST	______	○ 정상 ○ 비정상	○ 예 ○ 아니오
ALT	______	○ 정상 ○ 비정상	○ 예 ○ 아니오
ALP	______	○ 정상 ○ 비정상	○ 예 ○ 아니오
ɣGT	______	○ 정상 ○ 비정상	○ 예 ○ 아니오
LDH	______	○ 정상 ○ 비정상	○ 예 ○ 아니오
Total protein	______	○ 정상 ○ 비정상	○ 예 ○ 아니오
Albumin	______	○ 정상 ○ 비정상	○ 예 ○ 아니오
Globulin[자동계산]	______	○ 정상 ○ 비정상	○ 예 ○ 아니오

검사항목	결과	정상 여부	임상적 유의성
Ca	______	○ 정상 ○ 비정상	○ 예 ○ 아니오
Mg	______	○ 정상 ○ 비정상	○ 예 ○ 아니오
Na	______	○ 정상 ○ 비정상	○ 예 ○ 아니오
K	______	○ 정상 ○ 비정상	○ 예 ○ 아니오
Cl	______	○ 정상 ○ 비정상	○ 예 ○ 아니오
P	______	○ 정상 ○ 비정상	○ 예 ○ 아니오
Total cholesterol	______	○ 정상 ○ 비정상	○ 예 ○ 아니오
Triglyceride	______	○ 정상 ○ 비정상	○ 예 ○ 아니오
CPK	______	○ 정상 ○ 비정상	○ 예 ○ 아니오
Glucose	______	○ 정상 ○ 비정상	○ 예 ○ 아니오
CrCl[자동계산]	______		

12-Lead ECG	
ND	☐ ND
미실시인 경우 사유	______<br>______<br>______
검사일	______
측정횟수	○ 1회<br>○ 3회
BBB 여부	○ 예<br>○ 아니오

No.	Heart Rate	PR Interval	QRS Duration	QT Interval	QTcB [자동계산]	QTcF [자동계산]
1차	__ bpm	__ msec	__ msec	__ msec	__._	__._
2차	__ bpm	__ msec	__ msec	__ msec	__._	__._
3차	__ bpm	__ msec	__ msec	__ msec	__._	__._

QTcB 평균[자동계산]	__._
QTcF 평균[자동계산]	__._
임상적으로 유의한 비정상 소견이 있습니까?	○ 예<br>○ 아니오

* 동의서 취득 이후 임상적으로 유의한 비정상 소견이 있을 경우, 비정상 소견은 '이상반응'에 기록하십시오.
* Screen, C1D1 시점에서는 3회 측정하고, 이후 주기에서는 1회만 수행합니다.
(단, ECG를 통해 QT 간격 연장이 입증된 경우, 단기간(즉시)에 추가로 2개의 ECG를 수행하여 최종 3개 ECG의 평균 QTc interval을 확인합니다.)

ECHO 또는 MUGA scan	
ND	☐ ND
미실시인 경우 사유	____________________<br>____________________<br>____________________
검사일	______
검사 방법	○ ECHO<br>○ MUGA scan
좌심실구출률(LVEF)	__._ %
임상적으로 유의한 비정상 소견이 있습니까?	○ 예<br>○ 아니오
* 동의서 취득 이후 임상적으로 유의한 비정상 소견이 있을 경우, 비정상 소견은 '이상반응'에 기록하십시오.	

PK 채혈

ND	☐ ND
미실시인 경우 사유	____________________<br>____________________<br>____________________
PHI-101 투약일	______
PHI-101 투약시간	___

채혈 시점	ND	채혈일	채혈 시간
투여 전(-30분)	☐	______	___
투여 후 1~3시간	☐	______	___

방문일	
방문일	______

선행/병용약물 확인

지난 방문과 비교하였을 때, 새롭게 투여되었거나 투여 용량이 변경된 병용약물이 있는 경우 선행/병용약물 페이지에 기록하여 주십시오.

이상반응 확인

지난 방문과 비교하였을 때, 새롭게 발생한 이상반응이 있을 경우 이상반응 페이지에 기록하여 주십시오.
(만약, EOT방문 전 새로운 항암요법이 수행되는 경우에는 해당 시점까지만 이상반응을 수집합니다.)

**활력징후**	
ND	☐ ND
미실시인 경우 사유	______________________________<br>______________________________<br>______________________________
5분 이상 안정을 취한 후 앉은 자세(좌위)에서 측정합니다.	
측정일	______
수축기 혈압	__ mmHg
이완기 혈압	__ mmHg
맥박	__ 회/분
호흡수	__ 회/분
체온	__._ ℃
임상적으로 유의한 비정상 소견이 있습니까?<br>?	○ 예<br>○ 아니오
동의서 취득 이후 임상적으로 유의한 비정상 소견이 있을 경우, 비정상 소견은 ‘이상반응’에 기록하십시오.	

신체검진	
ND	☐ ND
미실시인 경우 사유	___
검진일	___
지난 방문과 비교하였을 때, 각 신체기관 검진 결과 새로운 비정상 소견이 있습니까?	○ 예 → 아래 표에 신체기관 및 상세내용을 기재하여 주십시오.<br>○ 아니오

동의서 취득 이후 임상적으로 유의한 비정상 소견이 있을 경우, 비정상 소견은 '이상반응'에 기록하십시오.

No	신체기관	임상적 유의성
1	___▼	○ 예 ○ 아니오

신체기관	외관 , 피부 , 두/경부 , 흉부/폐 , 심장 , 복부 , 비뇨/생식계 , 사지 , 근골격계 , 신경계 , 림프절 , 기타
신체기관_기타	___

ECOG PS	
ND	☐ ND
미실시인 경우 사유	______________________<br>______________________<br>______________________
평가일	______
ECOG PS	○ Grade 0<br>○ Grade 1<br>○ Grade 2<br>○ Grade 3<br>○ Grade 4<br>○ Grade 5

실험실 검사	
ND	☐ ND
미실시인 경우 사유	______
최소 8시간 금식 여부	○ 예<br>○ 아니오
검체 채취일	______

* 동의서 취득 이후 검사 결과가 'Clinical Significance' 일 경우 비정상 소견은 '이상반응'에 기록하십시오.
* 검사 항목별 단위는 "정상 참고 범위"와 함께 제공되므로 아래 결과에 기록하지 않습니다.

Hematology			
ND	☐ ND		
검사항목	결과	정상 여부	임상적 유의성
WBC	______	○ 정상 ○ 비정상	○ 예 ○ 아니오
RBC	______	○ 정상 ○ 비정상	○ 예 ○ 아니오
Hemoglobin	______	○ 정상 ○ 비정상	○ 예 ○ 아니오
Hematocrit	______	○ 정상 ○ 비정상	○ 예 ○ 아니오
Platelet	______	○ 정상 ○ 비정상	○ 예 ○ 아니오
MCV	______	○ 정상 ○ 비정상	○ 예 ○ 아니오
MCH	______	○ 정상 ○ 비정상	○ 예 ○ 아니오
MCHC	______	○ 정상 ○ 비정상	○ 예 ○ 아니오
Neutrophils	______	○ 정상 ○ 비정상	○ 예 ○ 아니오

검사항목	결과	정상 여부	임상적 유의성
Lymphocytes	______	○ 정상 ○ 비정상	○ 예 ○ 아니오
Monocytes	______	○ 정상 ○ 비정상	○ 예 ○ 아니오
Eosinophils	______	○ 정상 ○ 비정상	○ 예 ○ 아니오
Basophils	______	○ 정상 ○ 비정상	○ 예 ○ 아니오
ANC	______	○ 정상 ○ 비정상	○ 예 ○ 아니오

Chemistry			
ND	☐ ND		
검사항목	결과	정상 여부	임상적 유의성
BUN	______	○ 정상 ○ 비정상	○ 예 ○ 아니오
Creatinine	______	○ 정상 ○ 비정상	○ 예 ○ 아니오
Total bilirubin	______	○ 정상 ○ 비정상	○ 예 ○ 아니오
AST	______	○ 정상 ○ 비정상	○ 예 ○ 아니오
ALT	______	○ 정상 ○ 비정상	○ 예 ○ 아니오
ALP	______	○ 정상 ○ 비정상	○ 예 ○ 아니오
ɣGT	______	○ 정상 ○ 비정상	○ 예 ○ 아니오
LDH	______	○ 정상 ○ 비정상	○ 예 ○ 아니오
Total protein	______	○ 정상 ○ 비정상	○ 예 ○ 아니오
Albumin	______	○ 정상 ○ 비정상	○ 예 ○ 아니오
Globulin[자동계산]	______	○ 정상 ○ 비정상	○ 예 ○ 아니오

검사항목	결과	정상 여부	임상적 유의성
Ca	______	○ 정상 ○ 비정상	○ 예 ○ 아니오
Mg	______	○ 정상 ○ 비정상	○ 예 ○ 아니오
Na	______	○ 정상 ○ 비정상	○ 예 ○ 아니오
K	______	○ 정상 ○ 비정상	○ 예 ○ 아니오
Cl	______	○ 정상 ○ 비정상	○ 예 ○ 아니오
P	______	○ 정상 ○ 비정상	○ 예 ○ 아니오
Total cholesterol	______	○ 정상 ○ 비정상	○ 예 ○ 아니오
Triglyceride	______	○ 정상 ○ 비정상	○ 예 ○ 아니오
CPK	______	○ 정상 ○ 비정상	○ 예 ○ 아니오
Glucose	______	○ 정상 ○ 비정상	○ 예 ○ 아니오
CrCl[자동계산]	______		

12-Lead ECG

ND	☐ ND
미실시인 경우 사유	___<br>___<br>___
검사일	___
측정횟수	○ 1회<br>○ 3회
BBB 여부	○ 예<br>○ 아니오

No.	Heart Rate	PR Interval	QRS Duration	QT Interval	QTcB [자동계산]	QTcF [자동계산]
1차	__ bpm	__ msec	__ msec	__ msec	__.__	__.__
2차	__ bpm	__ msec	__ msec	__ msec	__.__	__.__
3차	__ bpm	__ msec	__ msec	__ msec	__.__	__.__

QTcB 평균[자동계산]	__.__
QTcF 평균[자동계산]	__.__
임상적으로 유의한 비정상 소견이 있습니까?	○ 예<br>○ 아니오

* 동의서 취득 이후 임상적으로 유의한 비정상 소견이 있을 경우, 비정상 소견은 ‘이상반응’에 기록하십시오.
* Screen, C1D1 시점에서는 3회 측정하고, 이후 주기에서는 1회만 수행합니다.
(단, ECG를 통해 QT 간격 연장이 입증된 경우, 단기간(즉시)에 추가로 2개의 ECG를 수행하여 최종 3개 ECG의 평균 QTc interval을 확인합니다.)

ECHO 또는 MUGA scan	
ND	☐ ND
미실시인 경우 사유	____________________<br>____________________<br>____________________
검사일	______
검사 방법	○ ECHO<br>○ MUGA scan
좌심실구출률(LVEF)	__. %
임상적으로 유의한 비정상 소견이 있습니까?	○ 예<br>○ 아니오
* 동의서 취득 이후 임상적으로 유의한 비정상 소견이 있을 경우, 비정상 소견은 ‘이상반응’에 기록하십시오.	

방문일	
방문일	______

선행/병용약물 확인

지난 방문과 비교하였을 때, 새롭게 투여되었거나 투여 용량이 변경된 병용약물이 있는 경우 선행/병용약물 페이지에 기록하여 주십시오.

이상반응 확인

지난 방문과 비교하였을 때, 새롭게 발생한 이상반응이 있을 경우 이상반응 페이지에 기록하여 주십시오.
(만약, EOT방문 전 새로운 항암요법이 수행되는 경우에는 해당 시점까지만 이상반응을 수집합니다.)

활력징후	
ND	☐ ND
미실시인 경우 사유	______________________<br>______________________<br>______________________
5분 이상 안정을 취한 후 앉은 자세(좌위)에서 측정합니다.	
측정일	______
수축기 혈압	__ mmHg
이완기 혈압	__ mmHg
맥박	__ 회/분
호흡수	__ 회/분
체온	__. ℃
임상적으로 유의한 비정상 소견이 있습니까?	○ 예<br>○ 아니오
동의서 취득 이후 임상적으로 유의한 비정상 소견이 있을 경우, 비정상 소견은 ‘이상반응’에 기록하십시오.	

신체계측	
ND	☐ ND
미실시인 경우 사유	______________________<br>______________________<br>______________________
측정일	______
체중	___. kg

신체검진	
ND	☐ ND
미실시인 경우 사유	________________________________<br>________________________________<br>________________________________
검진일	______
지난 방문과 비교하였을 때, 각 신체기관 검진 결과 새로운 비정상 소견이 있습니까?	○ 예 → 아래 표에 신체기관 및 상세내용을 기재하여 주십시오.<br>○ 아니오

동의서 취득 이후 임상적으로 유의한 비정상 소견이 있을 경우, 비정상 소견은 '이상반응'에 기록하십시오.

No	신체기관	임상적 유의성
1	___▼	○ 예 ○ 아니오

신체기관	외관 , 피부 , 두/경부 , 흉부/폐 , 심장 , 복부 , 비뇨/생식계 , 사지 , 근골격계 , 신경계 , 림프절 , 기타
신체기관_기타	________________________________<br>________________________________<br>________________________________

ECOG PS	
ND	☐ ND
미실시인 경우 사유	______<br>______<br>______
평가일	______
ECOG PS	○ Grade 0<br>○ Grade 1<br>○ Grade 2<br>○ Grade 3<br>○ Grade 4<br>○ Grade 5

임신검사	
ND	☐ ND
미실시인 경우 사유	________________<br>________________<br>________________
검사일	______
검사 방법	○ urine-hCG<br>○ serum-hCG
검사결과	○ 양성<br>○ 음성
urine-hCG(및/또는 serum hCG) 검사를 실시합니다.	

실험실 검사

ND	☐ ND
미실시인 경우 사유	______<br>______<br>______
최소 8시간 금식 여부	○ 예<br>○ 아니오
검체 채취일	______

* 동의서 취득 이후 검사 결과가 ‘Clinical Significance’ 일 경우 비정상 소견은 ‘이상반응’에 기록하십시오.
* 검사 항목별 단위는 “정상 참고 범위”와 함께 제공되므로 아래 결과에 기록하지 않습니다.

Hematology

ND	☐ ND

검사항목	결과	정상 여부	임상적 유의성
WBC	______	○ 정상 ○ 비정상	○ 예 ○ 아니오
RBC	______	○ 정상 ○ 비정상	○ 예 ○ 아니오
Hemoglobin	______	○ 정상 ○ 비정상	○ 예 ○ 아니오
Hematocrit	______	○ 정상 ○ 비정상	○ 예 ○ 아니오
Platelet	______	○ 정상 ○ 비정상	○ 예 ○ 아니오
MCV	______	○ 정상 ○ 비정상	○ 예 ○ 아니오
MCH	______	○ 정상 ○ 비정상	○ 예 ○ 아니오
MCHC	______	○ 정상 ○ 비정상	○ 예 ○ 아니오
Neutrophils	______	○ 정상 ○ 비정상	○ 예 ○ 아니오

검사항목	결과	정상 여부	임상적 유의성
Lymphocytes	______	○ 정상 ○ 비정상	○ 예 ○ 아니오
Monocytes	______	○ 정상 ○ 비정상	○ 예 ○ 아니오
Eosinophils	______	○ 정상 ○ 비정상	○ 예 ○ 아니오
Basophils	______	○ 정상 ○ 비정상	○ 예 ○ 아니오
ANC	______	○ 정상 ○ 비정상	○ 예 ○ 아니오

Chemistry			
ND	☐ ND		
검사항목	결과	정상 여부	임상적 유의성
BUN	______	○ 정상 ○ 비정상	○ 예 ○ 아니오
Creatinine	______	○ 정상 ○ 비정상	○ 예 ○ 아니오
Total bilirubin	______	○ 정상 ○ 비정상	○ 예 ○ 아니오
AST	______	○ 정상 ○ 비정상	○ 예 ○ 아니오
ALT	______	○ 정상 ○ 비정상	○ 예 ○ 아니오
ALP	______	○ 정상 ○ 비정상	○ 예 ○ 아니오
ɣGT	______	○ 정상 ○ 비정상	○ 예 ○ 아니오
LDH	______	○ 정상 ○ 비정상	○ 예 ○ 아니오
Total protein	______	○ 정상 ○ 비정상	○ 예 ○ 아니오
Albumin	______	○ 정상 ○ 비정상	○ 예 ○ 아니오
Globulin[자동계산]	______	○ 정상 ○ 비정상	○ 예 ○ 아니오

검사항목	결과	정상 여부	임상적 유의성
Ca	______	○ 정상 ○ 비정상	○ 예 ○ 아니오
Mg	______	○ 정상 ○ 비정상	○ 예 ○ 아니오
Na	______	○ 정상 ○ 비정상	○ 예 ○ 아니오
K	______	○ 정상 ○ 비정상	○ 예 ○ 아니오
Cl	______	○ 정상 ○ 비정상	○ 예 ○ 아니오
P	______	○ 정상 ○ 비정상	○ 예 ○ 아니오
Total cholesterol	______	○ 정상 ○ 비정상	○ 예 ○ 아니오
Triglyceride	______	○ 정상 ○ 비정상	○ 예 ○ 아니오
CPK	______	○ 정상 ○ 비정상	○ 예 ○ 아니오
Glucose	______	○ 정상 ○ 비정상	○ 예 ○ 아니오
CrCl[자동계산]	______		

Urinalysis			
ND	☐ ND		
검사항목	결과	정상 여부	임상적 유의성
Specific gravity	______	○ 정상 ○ 비정상	○ 예 ○ 아니오
pH	______	○ 정상 ○ 비정상	○ 예 ○ 아니오
Bilirubin[U]	______	○ 정상 ○ 비정상	○ 예 ○ 아니오
Urobilinogen[U]	______	○ 정상 ○ 비정상	○ 예 ○ 아니오
Blood[U]	______	○ 정상 ○ 비정상	○ 예 ○ 아니오

검사항목	결과	정상 여부	임상적 유의성
Glucose[U]	______	○ 정상 ○ 비정상	○ 예 ○ 아니오
Protein[U] (Alb[U])	______	○ 정상 ○ 비정상	○ 예 ○ 아니오
Ketones[U]	______	○ 정상 ○ 비정상	○ 예 ○ 아니오
Leukocytes[U]	______	○ 정상 ○ 비정상	○ 예 ○ 아니오

Thyroid Function Tests

ND	☐ ND		
검사항목	결과	정상 여부	임상적 유의성
TSH	______	○ 정상 ○ 비정상	○ 예 ○ 아니오
Free T4	______	○ 정상 ○ 비정상	○ 예 ○ 아니오

Tests for HbA1c

ND	☐ ND		
검사항목	결과	정상 여부	임상적 유의성
HbA1c	______	○ 정상 ○ 비정상	○ 예 ○ 아니오

Coagulation Profile

ND	☐ ND		
검사항목	결과	정상 여부	임상적 유의성
PT INR	______	○ 정상 ○ 비정상	○ 예 ○ 아니오
aPTT	______	○ 정상 ○ 비정상	○ 예 ○ 아니오

12-Lead ECG

ND	☐ ND
미실시인 경우 사유	______<br>______<br>______
검사일	______
측정횟수	○ 1회<br>○ 3회
BBB 여부	○ 예<br>○ 아니오

No.	Heart Rate	PR Interval	QRS Duration	QT Interval	QTcB [자동계산]	QTcF [자동계산]
1차	__ bpm	__ msec	__ msec	__ msec	__.	__.
2차	__ bpm	__ msec	__ msec	__ msec	__.	__.
3차	__ bpm	__ msec	__ msec	__ msec	__.	__.

QTcB 평균[자동계산]	__.
QTcF 평균[자동계산]	__.
임상적으로 유의한 비정상 소견이 있습니까?	○ 예<br>○ 아니오

* 동의서 취득 이후 임상적으로 유의한 비정상 소견이 있을 경우, 비정상 소견은 '이상반응'에 기록하십시오.
* Screen, C1D1 시점에서는 3회 측정하고, 이후 주기에서는 1회만 수행합니다.
(단, ECG를 통해 QT 간격 연장이 입증된 경우, 단기간(즉시)에 추가로 2개의 ECG를 수행하여 최종 3개 ECG의 평균 QTc interval을 확인합니다.)

ECHO 또는 MUGA scan	
ND	☐ ND
미실시인 경우 사유	______<br>______<br>______
검사일	______
검사 방법	○ ECHO<br>○ MUGA scan
좌심실구출률(LVEF)	__._ %
임상적으로 유의한 비정상 소견이 있습니까?	○ 예<br>○ 아니오
* 동의서 취득 이후 임상적으로 유의한 비정상 소견이 있을 경우, 비정상 소견은 '이상반응'에 기록하십시오.	

PK 채혈	
ND	☐ ND
미실시인 경우 사유	________________<br>________________<br>________________
PHI-101 투약일	______
PHI-101 투약시간	___

No	채혈일	채혈 시간
1	______	___

방문일	
방문일	______

선행/병용약물 확인
지난 방문과 비교하였을 때, 새롭게 투여되었거나 투여 용량이 변경된 병용약물이 있는 경우 선행/병용약물 페이지에 기록하여 주십시오.

이상반응 확인
지난 방문과 비교하였을 때, 새롭게 발생한 이상반응이 있을 경우 이상반응 페이지에 기록하여 주십시오.<br>(만약, EOT방문 전 새로운 항암요법이 수행되는 경우에는 해당 시점까지만 이상반응을 수집합니다.)

활력징후	
ND	☐ ND
미실시인 경우 사유	______________________<br>______________________<br>______________________
5분 이상 안정을 취한 후 앉은 자세(좌위)에서 측정합니다.	
측정일	______
수축기 혈압	__ mmHg
이완기 혈압	__ mmHg
맥박	__ 회/분
호흡수	__ 회/분
체온	__._ ℃
임상적으로 유의한 비정상 소견이 있습니까?	○ 예<br>○ 아니오
동의서 취득 이후 임상적으로 유의한 비정상 소견이 있을 경우, 비정상 소견은 '이상반응'에 기록하십시오.	

신체계측	
ND	☐ ND
미실시인 경우 사유	______________________<br>______________________<br>______________________
측정일	______
체중	___._ kg

신체검진	
ND	☐ ND
미실시인 경우 사유	________________<br>________________<br>________________
검진일	______
지난 방문과 비교하였을 때, 각 신체기관 검진 결과 새로운 비정상 소견이 있습니까?	○ 예 → 아래 표에 신체기관 및 상세내용을 기재하여 주십시오.<br>○ 아니오

동의서 취득 이후 임상적으로 유의한 비정상 소견이 있을 경우, 비정상 소견은 '이상반응'에 기록하십시오.

No	신체기관	임상적 유의성
1	___▼	○ 예 ○ 아니오

신체기관	외관 , 피부 , 두/경부 , 흉부/폐 , 심장 , 복부 , 비뇨/생식계 , 사지 , 근골격계 , 신경계 , 림프절 , 기타
신체기관_기타	________________<br>________________<br>________________

ECOG PS	
ND	☐ ND
미실시인 경우 사유	______<br>______<br>______
평가일	______
ECOG PS	○ Grade 0<br>○ Grade 1<br>○ Grade 2<br>○ Grade 3<br>○ Grade 4<br>○ Grade 5

임신검사	
ND	☐ ND
미실시인 경우 사유	______<br>______<br>______
검사일	______
검사 방법	○ urine-hCG<br>○ serum-hCG
검사결과	○ 양성<br>○ 음성
serum-hCG 검사 방법으로 실시합니다.	

실험실 검사	
ND	☐ ND
미실시인 경우 사유	______<br>______<br>______
최소 8시간 금식 여부	○ 예<br>○ 아니오
검체 채취일	______

* 동의서 취득 이후 검사 결과가 'Clinical Significance' 일 경우 비정상 소견은 '이상반응'에 기록하십시오.
* 검사 항목별 단위는 "정상 참고 범위"와 함께 제공되므로 아래 결과에 기록하지 않습니다.

Hematology			
ND	☐ ND		
검사항목	결과	정상 여부	임상적 유의성
WBC	______	○ 정상 ○ 비정상	○ 예 ○ 아니오
RBC	______	○ 정상 ○ 비정상	○ 예 ○ 아니오
Hemoglobin	______	○ 정상 ○ 비정상	○ 예 ○ 아니오
Hematocrit	______	○ 정상 ○ 비정상	○ 예 ○ 아니오
Platelet	______	○ 정상 ○ 비정상	○ 예 ○ 아니오
MCV	______	○ 정상 ○ 비정상	○ 예 ○ 아니오
MCH	______	○ 정상 ○ 비정상	○ 예 ○ 아니오
MCHC	______	○ 정상 ○ 비정상	○ 예 ○ 아니오
Neutrophils	______	○ 정상 ○ 비정상	○ 예 ○ 아니오

검사항목	결과	정상 여부	임상적 유의성
Lymphocytes	______	○ 정상 ○ 비정상	○ 예 ○ 아니오
Monocytes	______	○ 정상 ○ 비정상	○ 예 ○ 아니오
Eosinophils	______	○ 정상 ○ 비정상	○ 예 ○ 아니오
Basophils	______	○ 정상 ○ 비정상	○ 예 ○ 아니오
ANC	______	○ 정상 ○ 비정상	○ 예 ○ 아니오

Chemistry

ND	☐ ND		
검사항목	결과	정상 여부	임상적 유의성
BUN	______	○ 정상 ○ 비정상	○ 예 ○ 아니오
Creatinine	______	○ 정상 ○ 비정상	○ 예 ○ 아니오
Total bilirubin	______	○ 정상 ○ 비정상	○ 예 ○ 아니오
AST	______	○ 정상 ○ 비정상	○ 예 ○ 아니오
ALT	______	○ 정상 ○ 비정상	○ 예 ○ 아니오
ALP	______	○ 정상 ○ 비정상	○ 예 ○ 아니오
ɣGT	______	○ 정상 ○ 비정상	○ 예 ○ 아니오
LDH	______	○ 정상 ○ 비정상	○ 예 ○ 아니오
Total protein	______	○ 정상 ○ 비정상	○ 예 ○ 아니오
Albumin	______	○ 정상 ○ 비정상	○ 예 ○ 아니오
Globulin[자동계산]	______	○ 정상 ○ 비정상	○ 예 ○ 아니오

EOT	PROTOCOL	SITE	SCREENING NO.
실험실 검사	PHI-101-002		
전자서명			

검사항목	결과	정상 여부	임상적 유의성
Ca	______	○ 정상 ○ 비정상	○ 예 ○ 아니오
Mg	______	○ 정상 ○ 비정상	○ 예 ○ 아니오
Na	______	○ 정상 ○ 비정상	○ 예 ○ 아니오
K	______	○ 정상 ○ 비정상	○ 예 ○ 아니오
Cl	______	○ 정상 ○ 비정상	○ 예 ○ 아니오
P	______	○ 정상 ○ 비정상	○ 예 ○ 아니오
Total cholesterol	______	○ 정상 ○ 비정상	○ 예 ○ 아니오
Triglyceride	______	○ 정상 ○ 비정상	○ 예 ○ 아니오
CPK	______	○ 정상 ○ 비정상	○ 예 ○ 아니오
Glucose	______	○ 정상 ○ 비정상	○ 예 ○ 아니오
CrCl[자동계산]	______		

Urinalysis			
ND	☐ ND		
검사항목	결과	정상 여부	임상적 유의성
Specific gravity	______	○ 정상 ○ 비정상	○ 예 ○ 아니오
pH	______	○ 정상 ○ 비정상	○ 예 ○ 아니오
Bilirubin[U]	______	○ 정상 ○ 비정상	○ 예 ○ 아니오
Urobilinogen[U]	______	○ 정상 ○ 비정상	○ 예 ○ 아니오
Blood[U]	______	○ 정상 ○ 비정상	○ 예 ○ 아니오

검사항목	결과	정상 여부	임상적 유의성
Glucose[U]	______	○ 정상 ○ 비정상	○ 예 ○ 아니오
Protein[U] (Alb[U])	______	○ 정상 ○ 비정상	○ 예 ○ 아니오
Ketones[U]	______	○ 정상 ○ 비정상	○ 예 ○ 아니오
Leukocytes[U]	______	○ 정상 ○ 비정상	○ 예 ○ 아니오

Thyroid Function Tests

ND	☐ ND		
검사항목	결과	정상 여부	임상적 유의성
TSH	______	○ 정상 ○ 비정상	○ 예 ○ 아니오
Free T4	______	○ 정상 ○ 비정상	○ 예 ○ 아니오

Tests for HbA1c

ND	☐ ND		
검사항목	결과	정상 여부	임상적 유의성
HbA1c	______	○ 정상 ○ 비정상	○ 예 ○ 아니오

Coagulation Profile

ND	☐ ND		
검사항목	결과	정상 여부	임상적 유의성
PT INR	______	○ 정상 ○ 비정상	○ 예 ○ 아니오
aPTT	______	○ 정상 ○ 비정상	○ 예 ○ 아니오

12-Lead ECG

ND	☐ ND
미실시인 경우 사유	______<br>______<br>______
검사일	______
측정횟수	○ 1회<br>○ 3회
BBB 여부	○ 예<br>○ 아니오

No.	Heart Rate	PR Interval	QRS Duration	QT Interval	QTcB [자동계산]	QTcF [자동계산]
1차	__ bpm	__ msec	__ msec	__ msec	__._	__._
2차	__ bpm	__ msec	__ msec	__ msec	__._	__._
3차	__ bpm	__ msec	__ msec	__ msec	__._	__._

QTcB 평균[자동계산]	__._
QTcF 평균[자동계산]	__._
임상적으로 유의한 비정상 소견이 있습니까?	○ 예<br>○ 아니오

* 동의서 취득 이후 임상적으로 유의한 비정상 소견이 있을 경우, 비정상 소견은 '이상반응'에 기록하십시오.
* Screen, C1D1 시점에서는 3회 측정하고, 이후 주기에서는 1회만 수행합니다.
(단, ECG를 통해 QT 간격 연장이 입증된 경우, 단기간(즉시)에 추가로 2개의 ECG를 수행하여 최종 3개 ECG의 평균 QTc interval을 확인합니다.)

ECHO 또는 MUGA scan	
ND	☐ ND
미실시인 경우 사유	________________<br>________________<br>________________
검사일	______
검사 방법	○ ECHO<br>○ MUGA scan
좌심실구출률(LVEF)	__. %
임상적으로 유의한 비정상 소견이 있습니까 ?	○ 예<br>○ 아니오
* 동의서 취득 이후 임상적으로 유의한 비정상 소견이 있을 경우, 비정상 소견은 '이상반응'에 기록하십시오.	

PK 채혈	
ND	☐ ND
미실시인 경우 사유	______ ______ ______
PHI-101 투약일	______
PHI-101 투약시간	___

No	채혈일	채혈 시간
1	______	___

임상시험 투여종료	
시험대상자의 투여종료 시점	○ DLT 관찰기간(1주기)완료 전 DLT발생 없이 투여종료<br>○ DLT발생 또는 DLT 관찰기간(1주기) 이후 종료
종료/중단일	______
DLT 관찰기간(1주기)완료 전 DLT발생 없이 투여종료 사유	○ 영상학적 질병진행(radiographic progression)이 확인되거나 시험자가 치료효과부족(clinical progression)으로 판단한 경우<br>○ 대상자의 사망<br>○ 대상자 또는 대상자 대리인의 동의철회<br>○ 이상반응으로 임상시험 지속 곤란 (DLT제외) → 상세 내용 기재<br>○ 중대한 임상시험 계획서 위반 → 상세 내용 기재<br>○ 대상자가 지속적인 치료나 정규 방문에서 시행되는 검사 및 절차를 수행할 수 없는 상태나 다른 질환이 발견된 경우 → 상세 내용 기재<br>○ 추적관찰 실패<br>○ 기타 → 상세 내용 기재
DLT발생 또는 DLT 관찰기간(1주기) 이후 종료 사유	○ DLT 발생<br>○ 전체 임상시험 종료<br>○ PHI-101의 일시중단 기간이 4주 초과 또는 투여중지 2회 초과<br>○ 영상학적 질병진행(radiographic progression)이 확인되거나 시험자가 치료효과부족(clinical progression)으로 판단한 경우<br>○ 대상자의 사망<br>○ 대상자 또는 대상자 대리인의 동의철회<br>○ 이상반응으로 임상시험 지속 곤란 (DLT제외) → 상세 내용 기재<br>○ 중대한 임상시험 계획서 위반 → 상세 내용 기재<br>○ 대상자가 지속적인 치료나 정규 방문에서 시행되는 검사 및 절차를 수행할 수 없는 상태나 다른 질환이 발견된 경우 → 상세 내용 기재<br>○ 추적관찰 실패<br>○ 기타 → 상세 내용 기재
상세 내용	______<br>______<br>______

방문일	
방문일	______
방문사유	○ 이상반응<br>○ 기타
방문사유, 기타 상세	______<br>______<br>______

선행/병용약물 확인
지난 방문과 비교하였을 때, 새롭게 투여되었거나 투여 용량이 변경된 병용약물이 있는 경우 선행/병용약물 페이지에 기록하여 주십시오.

이상반응 확인
지난 방문과 비교하였을 때, 새롭게 발생한 이상반응이 있을 경우 이상반응 페이지에 기록하여 주십시오.<br>(만약, EOT방문 전 새로운 항암요법이 수행되는 경우에는 해당 시점까지만 이상반응을 수집합니다.)

활력징후	
ND	☐ ND
미실시인 경우 사유	______<br>______<br>______
5분 이상 안정을 취한 후 앉은 자세(좌위)에서 측정합니다.	
측정일	______
수축기 혈압	__ mmHg
이완기 혈압	__ mmHg
맥박	__ 회/분
호흡수	__ 회/분
체온	__. ℃
임상적으로 유의한 비정상 소견이 있습니까<br>?	○ 예<br>○ 아니오
동의서 취득 이후 임상적으로 유의한 비정상 소견이 있을 경우, 비정상 소견은 ‘이상반응’에 기록하십시오.	

신체계측	
ND	☐ ND
미실시인 경우 사유	______<br>______<br>______
측정일	______
체중	___. kg

신체검진	
ND	☐ ND
미실시인 경우 사유	___ ___ ___
검진일	___
지난 방문과 비교하였을 때, 각 신체기관 검진 결과 새로운 비정상 소견이 있습니까?	○ 예 → 아래 표에 신체기관 및 상세내용을 기재하여 주십시오.<br>○ 아니오

동의서 취득 이후 임상적으로 유의한 비정상 소견이 있을 경우, 비정상 소견은 ‘이상반응’에 기록하십시오.

No	신체기관	임상적 유의성
1	___▼	○ 예 ○ 아니오

신체기관	외관 , 피부 , 두/경부 , 흉부/폐 , 심장 , 복부 , 비뇨/생식계 , 사지 , 근골격계 , 신경계 , 림프절 , 기타
신체기관_기타	___ ___ ___

ECOG PS	
ND	☐ ND
미실시인 경우 사유	____________________<br>____________________<br>____________________
평가일	______
ECOG PS	○ Grade 0<br>○ Grade 1<br>○ Grade 2<br>○ Grade 3<br>○ Grade 4<br>○ Grade 5

임신검사	
ND	☐ ND
미실시인 경우 사유	________________<br>________________<br>________________
검사일	______
검사 방법	○ urine-hCG<br>○ serum-hCG
검사결과	○ 양성<br>○ 음성
urine-hCG(및/또는 serum hCG) 검사를 실시합니다.	

실험실 검사	
ND	☐ ND
미실시인 경우 사유	______<br>______<br>______
최소 8시간 금식 여부	○ 예<br>○ 아니오
검체 채취일	______

* 동의서 취득 이후 검사 결과가 'Clinical Significance' 일 경우 비정상 소견은 '이상반응'에 기록하십시오.
* 검사 항목별 단위는 "정상 참고 범위"와 함께 제공되므로 아래 결과에 기록하지 않습니다.

Hematology			
ND	☐ ND		
검사항목	결과	정상 여부	임상적 유의성
WBC	______	○ 정상 ○ 비정상	○ 예 ○ 아니오
RBC	______	○ 정상 ○ 비정상	○ 예 ○ 아니오
Hemoglobin	______	○ 정상 ○ 비정상	○ 예 ○ 아니오
Hematocrit	______	○ 정상 ○ 비정상	○ 예 ○ 아니오
Platelet	______	○ 정상 ○ 비정상	○ 예 ○ 아니오
MCV	______	○ 정상 ○ 비정상	○ 예 ○ 아니오
MCH	______	○ 정상 ○ 비정상	○ 예 ○ 아니오
MCHC	______	○ 정상 ○ 비정상	○ 예 ○ 아니오
Neutrophils	______	○ 정상 ○ 비정상	○ 예 ○ 아니오

검사항목	결과	정상 여부	임상적 유의성
Lymphocytes	______	○ 정상 ○ 비정상	○ 예 ○ 아니오
Monocytes	______	○ 정상 ○ 비정상	○ 예 ○ 아니오
Eosinophils	______	○ 정상 ○ 비정상	○ 예 ○ 아니오
Basophils	______	○ 정상 ○ 비정상	○ 예 ○ 아니오
ANC	______	○ 정상 ○ 비정상	○ 예 ○ 아니오

Chemistry			
ND	☐ ND		
검사항목	결과	정상 여부	임상적 유의성
BUN	______	○ 정상 ○ 비정상	○ 예 ○ 아니오
Creatinine	______	○ 정상 ○ 비정상	○ 예 ○ 아니오
Total bilirubin	______	○ 정상 ○ 비정상	○ 예 ○ 아니오
AST	______	○ 정상 ○ 비정상	○ 예 ○ 아니오
ALT	______	○ 정상 ○ 비정상	○ 예 ○ 아니오
ALP	______	○ 정상 ○ 비정상	○ 예 ○ 아니오
ɣGT	______	○ 정상 ○ 비정상	○ 예 ○ 아니오
LDH	______	○ 정상 ○ 비정상	○ 예 ○ 아니오
Total protein	______	○ 정상 ○ 비정상	○ 예 ○ 아니오
Albumin	______	○ 정상 ○ 비정상	○ 예 ○ 아니오
Globulin[자동계산]	______	○ 정상 ○ 비정상	○ 예 ○ 아니오

검사항목	결과	정상 여부	임상적 유의성
Ca	______	○ 정상 ○ 비정상	○ 예 ○ 아니오
Mg	______	○ 정상 ○ 비정상	○ 예 ○ 아니오
Na	______	○ 정상 ○ 비정상	○ 예 ○ 아니오
K	______	○ 정상 ○ 비정상	○ 예 ○ 아니오
Cl	______	○ 정상 ○ 비정상	○ 예 ○ 아니오
P	______	○ 정상 ○ 비정상	○ 예 ○ 아니오
Total cholesterol	______	○ 정상 ○ 비정상	○ 예 ○ 아니오
Triglyceride	______	○ 정상 ○ 비정상	○ 예 ○ 아니오
CPK	______	○ 정상 ○ 비정상	○ 예 ○ 아니오
Glucose	______	○ 정상 ○ 비정상	○ 예 ○ 아니오
CrCl[자동계산]	______		

Urinalysis			
ND	☐ ND		
검사항목	결과	정상 여부	임상적 유의성
Specific gravity	______	○ 정상 ○ 비정상	○ 예 ○ 아니오
pH	______	○ 정상 ○ 비정상	○ 예 ○ 아니오
Bilirubin[U]	______	○ 정상 ○ 비정상	○ 예 ○ 아니오
Urobilinogen[U]	______	○ 정상 ○ 비정상	○ 예 ○ 아니오
Blood[U]	______	○ 정상 ○ 비정상	○ 예 ○ 아니오

검사항목	결과	정상 여부	임상적 유의성
Glucose[U]	______	○ 정상 ○ 비정상	○ 예 ○ 아니오
Protein[U] (Alb[U])	______	○ 정상 ○ 비정상	○ 예 ○ 아니오
Ketones[U]	______	○ 정상 ○ 비정상	○ 예 ○ 아니오
Leukocytes[U]	______	○ 정상 ○ 비정상	○ 예 ○ 아니오

Thyroid Function Tests

ND	☐ ND		
검사항목	결과	정상 여부	임상적 유의성
TSH	______	○ 정상 ○ 비정상	○ 예 ○ 아니오
Free T4	______	○ 정상 ○ 비정상	○ 예 ○ 아니오

Tests for HbA1c

ND	☐ ND		
검사항목	결과	정상 여부	임상적 유의성
HbA1c	______	○ 정상 ○ 비정상	○ 예 ○ 아니오

Coagulation Profile

ND	☐ ND		
검사항목	결과	정상 여부	임상적 유의성
PT INR	______	○ 정상 ○ 비정상	○ 예 ○ 아니오
aPTT	______	○ 정상 ○ 비정상	○ 예 ○ 아니오

12-Lead ECG

ND	☐ ND
미실시인 경우 사유	______<br>______<br>______
검사일	______
측정횟수	○ 1회<br>○ 3회
BBB 여부	○ 예<br>○ 아니오

No.	Heart Rate	PR Interval	QRS Duration	QT Interval	QTcB [자동계산]	QTcF [자동계산]
1차	__ bpm	__ msec	__ msec	__ msec	__._	__._
2차	__ bpm	__ msec	__ msec	__ msec	__._	__._
3차	__ bpm	__ msec	__ msec	__ msec	__._	__._

QTcB 평균[자동계산]	__._
QTcF 평균[자동계산]	__._
임상적으로 유의한 비정상 소견이 있습니까?	○ 예<br>○ 아니오

* 동의서 취득 이후 임상적으로 유의한 비정상 소견이 있을 경우, 비정상 소견은 ‘이상반응’에 기록하십시오.
* Screen, C1D1 시점에서는 3회 측정하고, 이후 주기에서는 1회만 수행합니다.
(단, ECG를 통해 QT 간격 연장이 입증된 경우, 단기간(즉시)에 추가로 2개의 ECG를 수행하여 최종 3개 ECG의 평균 QTc interval을 확인합니다.)

ECHO 또는 MUGA scan	
ND	☐ ND
미실시인 경우 사유	____________<br>____________<br>____________
검사일	______
검사 방법	○ ECHO<br>○ MUGA scan
좌심실구출률(LVEF)	__. %
임상적으로 유의한 비정상 소견이 있습니까?	○ 예<br>○ 아니오
* 동의서 취득 이후 임상적으로 유의한 비정상 소견이 있을 경우, 비정상 소견은 '이상반응'에 기록하십시오.	

FU1-1	PROTOCOL	SITE	SCREENING NO.
방문일	PHI-101-002		
전자서명			

방문일	
방문일	______
방문유형	○ 기관 방문<br>○ 전화 방문

선행/병용약물 확인
지난 방문과 비교하였을 때, 새롭게 투여되었거나 투여 용량이 변경된 병용약물이 있는 경우 선행/병용약물 페이지에 기록하여 주십시오.

이상반응 확인
EOT 이후부터는 약물이상반응(adverse drug reaction, ADR)만 기재하여 주시기 바랍니다.<br>만약, EOT 방문 전 새로운 항암요법이 수행되는 경우에는 해당 시점 이후부터는 ADR만 수집합니다.)<br>* 약물이상반응(adverse drug reaction, ADR)이란 임상시험용 의약품의 임의 용량에서 발생한 모든 유해하고 의도하지 않은 반응으로써 임상시험용 의약품과의 인과관계를 부정할 수 없는 경우를 말합니다.

새로운 항암요법 및 생존여부 확인
* 새로운 항암요법을 시행한 경우, 새로운 항암 요법 확인 페이지에 기록하여 주십시오.<br>* 생존여부에 대한 정보를 추적 관찰하여 생존여부 확인 페이지에 기록하여 주십시오.<br>* 추적이 필요한 AE 및/또는 ADR이 있는 경우, 본 기간을 통해 수집될 수 있습니다.<br>* 해당하는 대상자가 있다면 종양반응 평가가 함께 진행될 수 있습니다. 평가진행 시, 종양평가 페이지에 기록하여 주십시오.

방문일	
방문일	______
방문유형	○ 기관 방문<br>○ 전화 방문

선행/병용약물 확인
지난 방문과 비교하였을 때, 새롭게 투여되었거나 투여 용량이 변경된 병용약물이 있는 경우 선행/병용약물 페이지에 기록하여 주십시오.

이상반응 확인
EOT 이후부터는 약물이상반응(adverse drug reaction, ADR)만 기재하여 주시기 바랍니다.<br>(만약, EOT 방문 전 새로운 항암요법이 수행되는 경우에는 해당 시점 이후부터는 ADR만 수집합니다.)<br>* 약물이상반응(adverse drug reaction, ADR)이란 임상시험용 의약품의 임의 용량에서 발생한 모든 유해하고 의도하지 않은 반응으로써 임상시험용 의약품과의 인과관계를 부정할 수 없는 경우를 말합니다.

새로운 항암요법 및 생존여부 확인
* 새로운 항암요법을 시행한 경우, 새로운 항암 요법 확인 페이지에 기록하여 주십시오.<br>* 생존여부에 대한 정보를 추적 관찰하여 생존여부 확인 페이지에 기록하여 주십시오.<br>* 추적이 필요한 AE 및/또는 ADR이 있는 경우, 본 기간을 통해 수집될 수 있습니다.<br>* 해당하는 대상자가 있다면 종양반응 평가가 함께 진행될 수 있습니다. 평가진행 시, 종양평가 페이지에 기록하여 주십시오.

PHI-101 투여

ND	☐ ND
미실시인 경우 사유	________________<br>________________<br>________________

No	주기	계획된 1일 복용량	계획된 투여량	실제 투여량	복약순응도
	Vomiting으로 미복용한 수량	투여 시작일	투여 종료일	중단 사유	
1	___▾	_ tab	_ tab	_ tab	__._ %
	_ tab	______	______	___▾	

주기	1주기 , 2주기 , 3주기 , 4주기 , 5주기 , 6주기 , 7주기 , 8주기 , 9주기 , 10주기 , 기타
주기_기타	________________<br>________________<br>________________
중단 사유	이상반응 , 기타
중단 사유_기타	________________<br>________________<br>________________

DLT 관찰	
DLT 평가 기간 동안 용량제한독성(DLT)이 발생하였습니까?	○ 예<br>○ 아니오<br>○ 평가불가
혈액학적 독성	☐ Febrile neutropenia (grade ≥ 3)<br>☐ Neutrophil count decreased (grade ≥ 3이면서, ＞7일인 경우)<br>☐ WBC decreased (grade ≥ 3)<br>☐ Platelet count decreased (grade ≥ 3 또는 grade ＜ 3이면서, 수혈을 요하는 경우)<br>☐ Anemia (grade ≥ 3)
비혈액학적 독성	☐ ECG QT corrected interval prolonged (grade ≥ 3)<br>☐ Nausea (grade ≥ 3이면서, 적절한 최적의 요법에도 불구하고 ＞ 7일인 경우)<br>☐ Tumor pain (grade ≥ 3이면서, 적절한 최적의 요법에도 불구하고 ＞ 7일인 경우)<br>☐ Vomiting (grade 3이면서, 적절한 최적의 요법에도 불구하고 ＞ 7일인 경우 또는 grade ≥ 4)<br>☐ Diarrhea or associated electrolyte abnormalities (grade ≥ 3이면서, 적절한 최적의 요법에도 불구하고 ＞ 2일인 경우)<br>☐ Fatigue (grade ≥ 3이면서, ＞ 7일인 경우)<br>☐ Anorexia (grade ≥ 3이면서, ＞ 7일인 경우)<br>☐ Hypophosphatemia, hypomagnesemia, or hypocalcemia (grade ≥ 3이면서, 적절한 최적의 요법에도 불구하고 ＞ 2일인 경우)<br>☐ 무증상의 AST, ALT, ALP or GGT (grade ≥ 3이면서, ＞ 7일인 경우)<br>☐ 간전이가 확인된 환자로서, 베이스라인 AST 또는 ALT ≥ 2.5~5 X ULN (AST 또는 ALT ＞ 8배 X ULN 상승 ＞ 7일인 경우)<br>☐ 간전이가 확인된 환자로서, 베이스라인 ALP ≥ 2~5 X ULN (ALP ＞ 8배 X ULN 상승 ＞ 7일인 경우)<br>☐ 1)~11)을 제외한 그 외 All ADR (grade ≥ 3)
기타 독성	☐ ADR이면서, PHI-101의 투여중지(일시중단) 기간 ＞ 4주인 경우

Comment	

임상시험 종료(EOS)	
임상시험 종료(EOS)방문 여부	○ 예<br>○ 아니오
EOS 종료일/중단일	______
임상시험 종료(EOS)방문 미실시 사유	○ 대상자의 사망<br>○ 추적관찰 실패<br>○ 대상자 또는 대상자 대리인의 동의철회<br>○ 기타 → Specify below
임상시험 종료 방문 미실시 사유, 기타	______<br>______<br>______

시험책임자 서명	
본 증례기록서의 모든 항목을 검토하였으며 빠짐없이 정확하게 기재하였음을 확인합니다.	
시험책임자 서명	______
서명일	________________

방문일

방문일	______
방문유형	○ 기관 방문<br>○ 전화 방문

선행/병용약물 확인

지난 방문과 비교하였을 때, 새롭게 투여되었거나 투여 용량이 변경된 병용약물이 있는 경우 선행/병용약물 페이지에 기록하여 주십시오.

이상반응 확인

EOT 이후부터는 약물이상반응(adverse drug reaction, ADR)만 기재하여 주시기 바랍니다.
만약, EOT 방문 전 새로운 항암요법이 수행되는 경우에는 해당 시점 이후부터는 ADR만 수집합니다.)
* 약물이상반응(adverse drug reaction, ADR)이란 임상시험용 의약품의 임의 용량에서 발생한 모든 유해하고 의도하지 않은 반응으로써 임상시험용 의약품과의 인과관계를 부정할 수 없는 경우를 말합니다.

새로운 항암요법 및 생존여부 확인

* 새로운 항암요법을 시행한 경우, 새로운 항암 요법 확인 페이지에 기록하여 주십시오.
* 생존여부에 대한 정보를 추적 관찰하여 생존여부 확인 페이지에 기록하여 주십시오.
* 추적이 필요한 AE 및/또는 ADR이 있는 경우, 본 기간을 통해 수집될 수 있습니다.
* 해당하는 대상자가 있다면 종양반응 평가가 함께 진행될 수 있습니다. 평가진행 시, 종양평가 페이지에 기록하여 주십시오.

CA 125 검사

ND	☐ ND

* CA 125 검사 일정은 달력일(calendar day)기준으로 C1D1 이후 8주마다 계획되며, 주기 시작 지연 등에 따른 조정을 해서는 안됩니다.
 CA 125 검사일은 계획된 검사일의 ±1주일 내에서 대상자의 병원 방문일정에 맞춰 방문 가능합니다.
*베이스라인(C1D1)을 기준으로 28일 이내 수행된 검사 내용이 없는 경우, 스크리닝 시 수행합니다.
*EOT 방문 전 4주(28일) 이내 확보된 검사가 있다면, EOT 방문의 검사는 생략 가능합니다.

No	ND	미실시 사유	검진일	결과	정상 여부	임상적 유의성
1	☐	___________<br>___________<br>___________	______	______	○ 정상<br>○ 비정상	○ 예 ○ 아니오

종양평가

ND	☐ ND

* 베이스라인(C1D1)을 기준으로 28일 이내 수행된 CT scan (abdomen, pelvis, chest 등) 또는 MRI 검사 내용이 없는 경우, 스크리닝 시 수행합니다.
* CT scan 조영제에 대한 과민반응의 이력 또는 과민반응이 있는 경우, 시험자 판단에 따라 MRI로 수행될 수 있습니다.
* 매 평가 시점과 베이스라인(C1D1)에서의 영상검사 방법은 동일해야 합니다.
* 종양반응 평가 일정은 달력일(calendar day)기준으로 C1D1 이후 8주마다 계획되며, 주기 시작 지연 등에 따른 조정을 해서는 안됩니다.
 CT scan(또는 MRI) 측정일은 계획된 종양반응 평가일의 ±1주일 내에서 대상자의 병원 방문일정에 맞춰 방문 가능합니다.
* EOT 방문 전 4주(28일) 이내 확보된 검사가 있다면, EOT 방문의 검사는 생략 가능합니다.
* EOT 방문 시까지 PD가 확인되지 않은 대상자는 'EOS 시점, PD 확인 시점, 새로운 항암요법의 시작, 동의철회 또는 사망' 중 선행되는 사건이 확인될 때까지 8주(±1주)마다 종양반응 평가를 실시합니다.

No	ND	Target Lesion Response by RECIST	Non-target Lesion Response by RECIST	New Lesion	Overall Response by RECIST

종양평가 #1	
No	1
ND	☐ ND
미실시인 경우 사유	______<br>______<br>______
Date of Measurement	______
Method	___▼

Method	CT , MRI , Other
Method_Other	______<br>______<br>______

Target Lesion	
None	☐ None
Target Lesion 개수	___▼

Target Lesion 개수	1 , 2 , 3 , 4 , 5

Lesion	Organ	Detailed Location	Lymph Node	Size(mm)	Comment
1	______<br>______<br>______	______<br>______<br>______	○ Yes ○ No	__.__	______<br>______<br>______
2	______<br>______<br>______	______<br>______<br>______	○ Yes ○ No	__.__	______<br>______<br>______

Lesion	Organ	Detailed Location	Lymph Node	Size(mm)	Comment
3	______ ______ ______	______ ______ ______	○ Yes ○ No	__.	______ ______ ______
4	______ ______ ______	______ ______ ______	○ Yes ○ No	__.	______ ______ ______
5	______ ______ ______	______ ______ ______	○ Yes ○ No	__.	______ ______ ______

Sum of Size(mm)[자동계산]	__.
Change from Baseline[자동계산]	__. %
Change from Nadir[자동계산]	__. %
Target Lesion Response by RECIST	○ CR ○ PR ○ SD ○ PD ○ NE
**Non-target Lesion**	
None	☐ None
Non-Target Lesion 개수	___▼

Non-Target Lesion 개수	1 , 2 , 3 , 4 , 5

Lesion	Organ	Detailed Location	Lymph Node	Tumor Status	Comment
1	______ ______ ______	______ ______ ______	○ Yes ○ No	___▼	______ ______ ______

Lesion	Organ	Detailed Location	Lymph Node	Tumor Status	Comment
2	______<br>______<br>______	______<br>______<br>______	○ Yes ○ No	___▼	______<br>______<br>______
3	______<br>______<br>______	______<br>______<br>______	○ Yes ○ No	___▼	______<br>______<br>______
4	______<br>______<br>______	______<br>______<br>______	○ Yes ○ No	___▼	______<br>______<br>______
5	______<br>______<br>______	______<br>______<br>______	○ Yes ○ No	___▼	______<br>______<br>______

Tumor Status	Present , Absent , Unequivocal Progression
Tumor Status	Present , Absent , Unequivocal Progression
Tumor Status	Present , Absent , Unequivocal Progression
Tumor Status	Present , Absent , Unequivocal Progression
Tumor Status	Present , Absent , Unequivocal Progression

Non-target Lesion Response by RECIST	○ CR ○ Non-CR/Non-PD ○ PD ○ NE

New Lesion

New Lesion	○ 예 → 아래에 기록하십시오.<br>○ 아니오

'예' 일 경우 전반적 반응은 질병의 진행(PD)에 해당합니다.

Lesion	Organ	Detailed Location	Lymph Node	Comment
1			○ Yes ○ No	

Lesion	Organ	Detailed Location	Lymph Node	Comment
	______<br>______<br>______	______<br>______<br>______		______<br>______<br>______

Overall Response - RECIST	
Date of Evaluation	______
Overall Response by RECIST	○ Complete response (CR)<br>○ Partial response (PR)<br>○ Stable disease (SD)<br>○ Progression (PD)<br>○ Not Evaluable(NE) → Specify reason below
NE reason	______<br>______<br>______

Best Overall Response - RECIST	
NA	☐ NA
Date of Evaluation	______
Best Overall Response by RECIST	○ Complete response (CR)<br>○ Partial response (PR)<br>○ Stable disease (SD)<br>○ Progression (PD)<br>○ Not Evaluable(NE) → Specify reason below
NE reason	______<br>______<br>______

이상반응

None	☐ None

* 이상반응은 동의서 취득 이후부터 PHI-101의 마지막 투여일(EOT)까지 기재하여 주시기 바랍니다.
* EOT 이후부터는 ADR만 기재하여 주시기 바랍니다. (만약, EOT 방문 전 새로운 항암요법이 수행되는 경우에는 해당 시점 이후부터는 ADR만 수집합니다)
* 이상반응으로 인해 PHI-101투여가 변경/중단되는 경우, [PHI-101 투여] 페이지의 [중단 사유]에 해당 내용을 함께 기재하여 주시기 바랍니다.
* 치료여부에 약물/비약물 치료를 체크하는 경우, [선행/병용요법 (약물)] 혹은 [선행/병용요법 (비약물)] 페이지에 해당 치료 내용을 함께 기재하여 주시기 바랍니다.

No	이상반응명	TEAE	발생일	중증도	결과	소실일	SAE	인과관계	관련 조치	치료 여부
1	______<br>______<br>______	☐	______	___▼	___▼	______	___▼	___▼	___▼	___▼

SAE	SAE 아님 , SAE, 사망 , SAE, 생명을 위협 , SAE, 입원 또는 입원 기간의 연장 , SAE, 지속적 또는 의미 있는 불구나 기능저하 , SAE, 선천성 기형 또는 이상 초래 , SAE, 그 밖에 의학적으로 중요한 상황이 발생하는 사례
인과관계	관련성이 명백함 , 관련성이 많음 , 관련성이 의심됨 , 관련성이 적음 , 평가 불가능 , 관련성이 없음
관련 조치	일시중단 , 영구중단 , 용량감량 , 용량증량 , 용량변화 없음 , 알 수 없음 , 해당사항 없음
치료 여부	약물 치료 , 비약물 치료 , 약물치료 및 비약물 치료 , 처치를 시행하지 않음
중증도	Grade1(경증) , Grade2(중등증) , Grade3(중증) , Grade4(생명을 위협) , Grade5(사망)
결과	회복(해결)됨 , 회복(해결)중임 , 회복(해결)되지 않음 , 회복(해결)되었으나 후유증이 남음 , 사망 , 알 수 없음

선행/병용약물

None	☐ None

* Screen방문 기준 24주 이내의 약물치료 기록을 모두 기재하십시오.
(단, 선정/제외 기준과 관련된 요법의 경우, 계획서에 따라 검토되어야 하는 기간에 준하여 모두 조사한다.)
* 용량, 단위, 투여목적 등을 정확히 기재하십시오.
* 해당 약물이 투여 중인 경우 ‘지속중’에 체크하십시오.
* 현재 진행중인 병력 혹은 이상반응으로 인한 약제 투여일 경우 해당 병력 및 이상반응을 투여목적에 기재하십시오
.
* EOT 이전 투여된 새로운 항암요법이 있는 경우 새로운 항암 요법 확인 페이지에 기재하십시오.

No	약제명	1일 투여량	단위	경로
	투여 시작일	지속중	투여 종료일	투여목적
1	______	______	______	______
	______	☐	______	______▼

투여목적	병력 , 이상반응 , 기타
투여목적_병력	______
투여목적_이상반응	______
투여목적_기타	______

선행/병용요법

None	☐ None

* Screen 방문 기준 24주 이내의 비약물치료 기록을 모두 기재하십시오.
(단, 선정/제외 기준과 관련된 요법의 경우, 계획서에 따라 검토되어야 하는 기간에 준하여 모두 조사하십시오.)
* 해당 치료를 받는 중인 경우 '지속중'에 체크하십시오.
* 현재 진행중인 병력 혹은 이상반응으로 인한 치료일 경우 해당 병력 및 이상반응을 투여목적에 기재하십시오.
* 임상약 투여 이후 시행된 방사선요법이 있는 경우 본 페이지에 기재하며, 치료명에 방사선 조사 부위를 함께 기재하십시오.

No	치료명	치료시작일	지속중	치료종료일	치료목적
1	______<br>______<br>______	______	☐	______	___▼

치료목적	병력 , 이상반응 , 기타
치료목적_병력	______<br>______<br>______
치료목적_이상반응	______<br>______<br>______
치료목적_기타	______<br>______<br>______

새로운 항암 요법 확인	
새로운 항암요법을 시행하였습니까?	○ 예<br>○ 아니오<br>○ 알 수 없음
‘알 수 없음’인 경우 사유	______________________<br>______________________<br>______________________

No	시작시점	새로운 항암요법명	시작일
1	○ EOT이전<br>○ EOT이후	__________<br>__________<br>__________	______

생존여부 확인

None	☐ None

No	확인 시점	날짜	생존여부	알 수 없음, 사유	사망일	사망원인	사망원인, 기타
1	○ EOT~EOS사이 매8주<br>○ EOS이후 매12주	______	○ 예<br>○ 아니오<br>○ 알 수 없음	____________<br>____________<br>____________	______	○ 질병의 진행<br>○ 이상반응<br>○ 기타	____________<br>____________<br>____________

임상시험 종료(FU2)	
임상시험 종료(EOS)방문 이후 1년동안 추적관찰 종료 여부	○ 예<br>○ 아니오
추적관찰종료/중단일	______
추적관찰 종료 중단 사유	○ 대상자의 사망<br>○ 추적관찰 실패<br>○ 대상자 또는 대상자 대리인의 동의철회<br>○ 기타 → Specify below
추적관찰 종료 중단 사유, 기타	______<br>______<br>______

시험책임자 서명	
본 증례기록서의 모든 항목을 검토하였으며 빠짐없이 정확하게 기재하였음을 확인합니다.	
시험책임자 서명	______
서명일	__________________
